# The P-type pentatricopeptide repeat protein DWEORG1 is a non-previously reported rPPR protein of Arabidopsis mitochondria

**DOI:** 10.1038/s41598-022-16812-0

**Published:** 2022-07-21

**Authors:** Stefanie Grüttner, Tan-Trung Nguyen, Anika Bruhs, Hakim Mireau, Frank Kempken

**Affiliations:** 1 Abteilung Botanische Genetik und Molekularbiologie, Botanisches Institut und Botanischer Garten, Christian-Albrechts-Universität zu Kiel, Olshausenstraße 40, 24098 Kiel, Germany; 2 Institut Jean-Pierre Bourgin INRA, AgroParisTech, CNRS, Université Paris-Saclay, Versailles, France

**Keywords:** Molecular biology, Plant sciences

## Abstract

Gene expression in plant mitochondria is mainly regulated by nuclear-encoded proteins on a post-transcriptional level. Pentatricopeptide repeat (PPR) proteins play a major role by participating in mRNA stability, splicing, RNA editing, and translation initiation. PPR proteins were also shown to be part of the mitochondrial ribosome (rPPR proteins), which may act as regulators of gene expression in plants. In this study, we focus on a mitochondrial-located P-type PPR protein—DWEORG1—from *Arabidopsis thaliana*. Its abundance in mitochondria is high, and it has a similar expression pattern as rPPR proteins. Mutant *dweorg1* plants exhibit a slow-growth phenotype. Using ribosome profiling, a decrease in translation efficiency for *cox2*, *rps4*, *rpl5*, and *ccmF*_*N2*_ was observed in *dweorg1* mutants, correlating with a reduced accumulation of the Cox2 protein in these plants. In addition, the mitochondrial rRNA levels are significantly reduced in *dweorg1* compared with the wild type. DWEORG1 co-migrates with the ribosomal proteins Rps4 and Rpl16 in sucrose gradients, suggesting an association of DWEORG1 with the mitoribosome. Collectively, this data suggests that *DWEORG1* encodes a novel rPPR protein that is needed for the translation of *cox2*, *rps4*, *rpl5*, and *ccmF*_*N2*_ and provides a stabilizing function for mitochondrial ribosomes.

## Introduction

Given their endosymbiotic origin, mitochondria harbor their own genome and therefore a complete gene expression system^1^. During the process of mitochondrial evolution, most of the prokaryotic ancestor genes were either lost or transferred to the nucleus, creating nuclear-encoded genes whose encoded proteins became important for mitochondrial functions. Meanwhile, a protein import machinery into the mitochondria was established^2,3^. Furthermore, mitochondria and the nucleus communicate via retrograde signaling to regulate the expression of nuclear-encoded mitochondrial genes in response to mitochondrial activity^4^. Mitochondrial genomes have highly reduced coding capacities that are insufficient for mitochondrial gene expression and regulation. Mitochondrial gene expression is controlled by nuclear-encoded mitochondrial proteins, such as pentatricopeptide repeat (PPR) proteins, a particularly large protein family involved in the expression of mitochondrial genes^5^.

More than 450 PPR proteins are encoded in *Arabidopsis thaliana*^6^. They are characterized by degenerated 31–36 amino acid tandem repeats, which may occur as 4–20 copies along the proteins^7,8^. PPR proteins are divided into two subclasses: the P-class, containing only the canonical 35-amino acid P-motif; and the PLS-class, having additional longer and shorter motifs (the L and S motifs). The PLS-class proteins may carry additional C-terminal E1, E2 or DYW domains^9^. The PPR motif folds into two antiparallel α-helices, and the repeats form a superhelix containing a central grove. This structure allows PPR proteins to bind an RNA target in a sequence-specific manner that is mostly dictated by the amino acids at positions 5 and 35 of each PPR repeat^9–11^. Almost all PPR proteins are found in mitochondria and chloroplasts where they are involved in most steps of organellar post-transcriptional processes. PLS-class proteins are almost exclusively involved in RNA editing in mitochondria and chloroplasts^12–14^, while P-class PPR proteins are involved in intron splicing, 5′- and 3′-end maturation, RNA stability, and mRNA translation^5–7^. Recently, P-class PPR proteins (called rPPR proteins) were also found to be subunits of plant mitochondrial ribosomes (mitoribosomes)^15,16^.

Mitochondrial gene expression is a complex mechanism that is, so far, only poorly understood. It is mostly controlled via post-transcriptional, translational, and post-translational rather than transcriptional processes. Hence, the mitochondrial mRNA level does not always correlate with the actual protein level^17–20^. With the exception of *Reclinomonas americana*, mitochondrial transcripts do not possess a Shine–Dalgarno or Shine-Dalgarno-like sequence to help in the recruitment of the translation initiation machinery near AUG start codons, as their prokaryotic ancestors or chloroplasts do^21–23^. Moreover, the 5′-untranslated regions (UTRs) in mitochondrial transcripts differ greatly from one another, and a few mRNAs encoding dicistronic transcripts exist^24^. In contrast to yeast and humans, to this day, how the plant mitochondrial translation machinery detects translation start codons and how the plant mitochondrial translation is regulated is still not understood. Although such translational regulation in plant mitochondria is yet to be thoroughly studied, evidence suggests that it is at play. Higher ribosome loading and therefore a higher rate of translation is observed for mRNAs of the oxidative phosphorylation (OXPHOS) chain complexes in comparison to other mitochondria-encoded mRNAs^25,26^. In addition, a defect in the ribosomal protein RPS10 leads to a change in translational activity in plant mitochondria where the OXPHOS components are less translated and the translation efficiency of mRNAs for ribosomal proteins, the intron maturase MatR and the transport membrane protein MttB increases^27^. Some studies have suggested that ribosomal recruitment is regulated via gene-specific sequences and trans-factors such as PPR proteins in plant mitochondria^28,29^. In addition to the translational regulation, post-translational regulation mechanisms can be observed in plant mitochondria. For instance, unedited transcripts were found to be associated with translating ribosomes^25^, but unedited protein products are generally not detected in plant mitochondria^30,31^. Hence, protein products deriving from non-edited transcripts must be degraded post-translationally. Similarly, a regulation on protein levels of the OXPHOS chain components is observed as non-assembled excessive subunits are degraded by ATP-dependent proteases^32–34^. Recently, the structure and composition of plant mitochondrial ribosomes were described^15,16^. They consist of two subunits: the large subunit and the small subunit (LSU and SSU, respectively), both of which are ribonucleoprotein complexes with a distinct combination of rRNAs and proteins. All of the mitoribosome rRNAs are encoded in the mitochondria, while most of the ribosomal proteins are encoded in the nucleus^15,16,35^. The plant mitoribosome is determined as one of the largest and most complex ribosomes^15^, while the LSU of plant mitoribosomes is comparable with that of animals the SSU largely differs in size and shape. The rRNAs of the SSU and LSU in plant mitoribosomes are 20% and 9% larger than those in *Escherichia coli*, respectively. They contain several expansion segments (ESs)—370 nt in helix 39 (h39), 56 nt in h6, and 47 nt in h44 of the SSU 18S Rrna—and several short insertions in the LSU 26S rRNA’s domain III in helices H52-H55. The SSU in plant mitoribosomes contains a distinctive foot extension that stems from the h44 ES, body protuberance, and a very large head as a result of the h39 ES. In contrast to all other as yet described mitoribosomes, the LSU of plant mitoribosomes harbors a 5S rRNA in the central protuberance^36^. While the rRNA content of plant mitoribosomes therefore exceeds that of many other mitoribosomes, the protein content (45 LSU and 37 SSU proteins) is similar to those of others, with only 19 plant-specific ribosomal proteins, of which 11 belong to the PPR family^15,16,36^. The ribosomal PPR proteins most likely function in stabilizing the plant mitoribosome-specific rRNA ESs and might be involved in translation initiation or the attachment of the mitoribosomes to the inner mitochondrial membrane^36^.

In this study, we identified a mitochondrial-localized P-type PPR protein, DWEORG1, that is associated with mitoribosomes in *A. thaliana*. Mutant plants show a global decrease of mitochondrial translational efficiency and a significant reduction in mitochondrial rRNA stability, suggesting a destabilization of mitoribosomes.

## Results

### DWEORG1 is a P-type PPR protein targeted to mitochondria with a similar expression pattern to rPPR proteins

**Figure 1:**
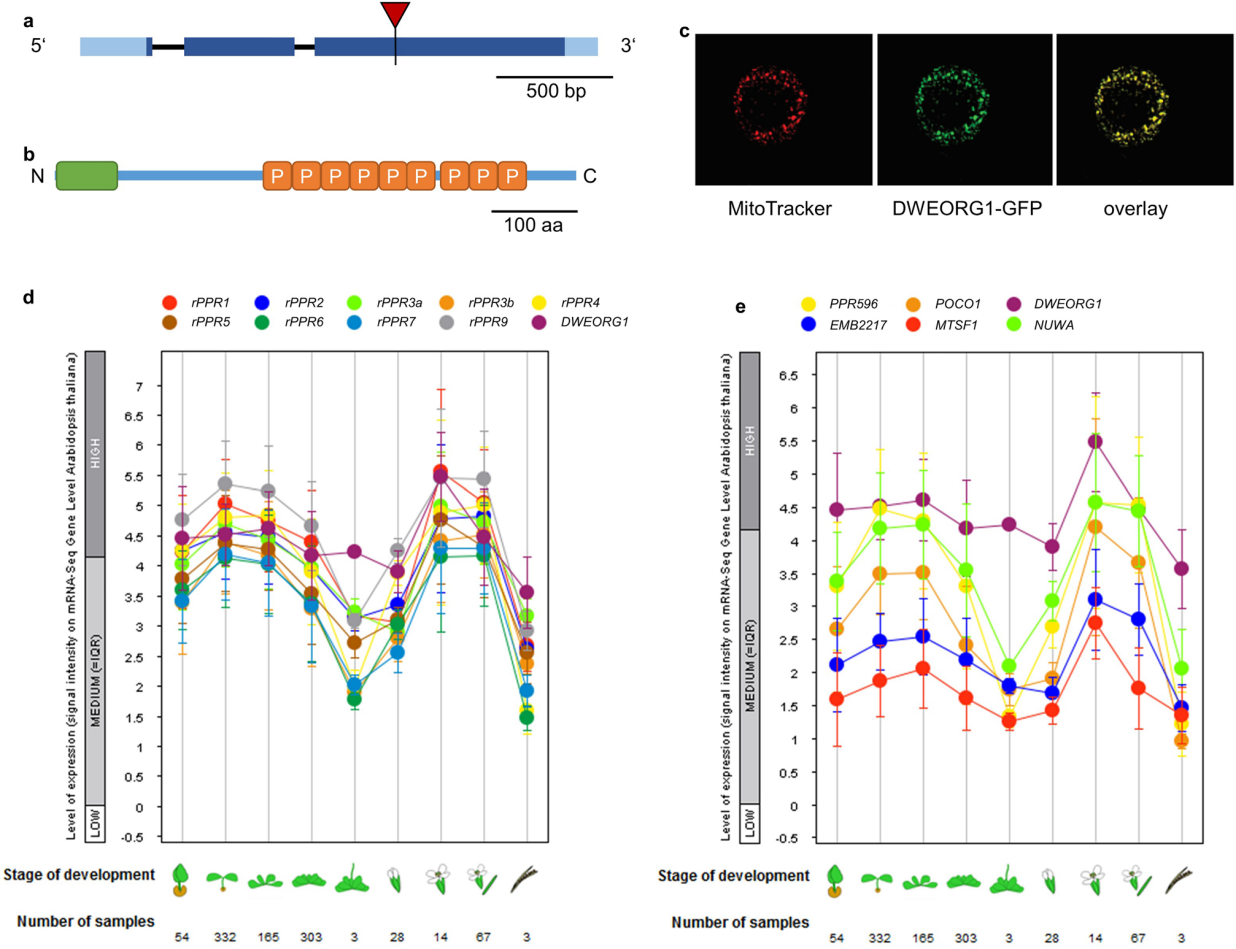
*dweorg1* encodes a mitochondria-targeted PPR protein, and its expression profile is highly similar to that of ribosomal PPR genes. (**a**) *dweorg1* gene structure and position of the T-DNA insertion site in the third exon (red triangle). Exons and introns are shown as dark blue boxes and black lines, respectively. UTRs are depicted in light blue. (**b**) DWEORG1 protein structure. The mitochondrial signal peptide, predicted by TargetP, is shown as a green box. The P-type PPR motifs are shown as orange boxes (P). (**c**) Confocal laser scanning microscopy images of an *Arabidopsis thaliana* leaf protoplast transiently expressing a DWEORG1–eGFP fusion protein (green signal) and also stained with the MitoTracker mitochondrial marker (red signal). The GFP signal and MitoTracker signals overlap perfectly (yellow). (**d**) Expression patterns of *rPPR1*, *rPPR2*, *rPPR3a*, *rPPR3b*, *rPPR4*, *rPPR5*, *rPPR6*, *rPPR7*, and *rPPR9* genes throughout *Arabidopsis* development in comparison with that of *DWEORG1*. The figure was made with GENEVESTIGATOR^40^. (**e**) Expression pattern of mitochondrially located P-type PPR protein genes *PPR596*, *POCO1*, *EMB2217*, *MTSF1*, and *NUWA* throughout *Arabidopsis* development in comparison with that of *DWEORG1*. The figure was made with GENEVESTIGATOR^40^.

The composition of the *Arabidopsis* mitoribosome revealed the presence of 11 ribosomal PPR proteins whose accumulation levels are elevated and identical to other mitoribosomal subunits^15,16,36^. Such high production levels are highly divergent from most mitochondria-targeted PPR proteins that correspond to poorly abundant mRNA-specific trans-factors^7^. However, several other PPR proteins that are predicted to target mitochondria accumulate similarly to rPPR proteins but were not reported to be part of the *Arabidopsis* mitoribosome^37^. To determine the role that such PPR proteins could fulfill, we characterized the function of the AT3G15590 PPR protein. A recent analysis of the mitochondrial proteome^37^ showed that the AT3G15590 protein is an abundant P-type PPR protein with 926 copies/mitochondrion, similar to the abundance of rPPR proteins (442 to 2,189 copies/mitochondrion). The gene *AT3G15590* of *A. thaliana* consists of three exons and two introns (Fig. 1a). Its open reading frame codes for a PPR protein of 610 amino acids in length and a molecular weight of 69.39 kDa. This PPR protein contains nine P-type PPR motifs and no additional C-terminal domains. The AlphaFold prediction^38,39^ of DWEORG1 shows repeats consisting of pairs of antiparallel α-helices matching the typical structure of PPR proteins^7,8^ (Supplementary Fig. S1). Using TargetP (http://www.cbs.dtu.dk/services/TargetP), a putative mitochondrial localization signal was predicted at the N-terminal region of AT3G15590 (Fig. 1b).

Its mitochondrial localization was confirmed by transforming *Arabidopsis* leaf protoplasts with a DWEORG1-eGFP fusion protein. The mitochondria were stained with MitoTracker, and the transformed protoplasts were analyzed with a confocal laser scanning microscope. The red MitoTracker signal overlapped with the GFP fluorescence of the fusion protein (Fig. 1c), confirming the mitochondrial localization of AT3G15590.

Since the abundance of AT3G15590 resembles that of rPPR proteins, the transcription pattern of its gene was compared with that of rPPRs and other kinds of P-type PPRs using GENEVESTIGATOR^40^. The AT3G15590 gene shows a high expression pattern during all developmental stages of *Arabidopsis*, with a peak in reproductive tissues, very similar to that of known rPPR proteins (Fig. 1d). The only difference to rPPRs is seen in the phase of stem elongation, where the expression of *DWEORG1* exceeds that of the rPPR genes. Compared with other P-type PPR protein genes, the expression pattern of AT3G15590 differed by showing a generally higher expression level at all the *Arabidopsis* developmental stages (Fig. 1e). Overall, this suggests that the function of AT3G15590 could be related to rPPR proteins, although it was not found to be part of the *Arabidopsis* mitoribosome^15,16^.

### *Dweorg1* plants show a dwarf phenotype

**Figure 2:**
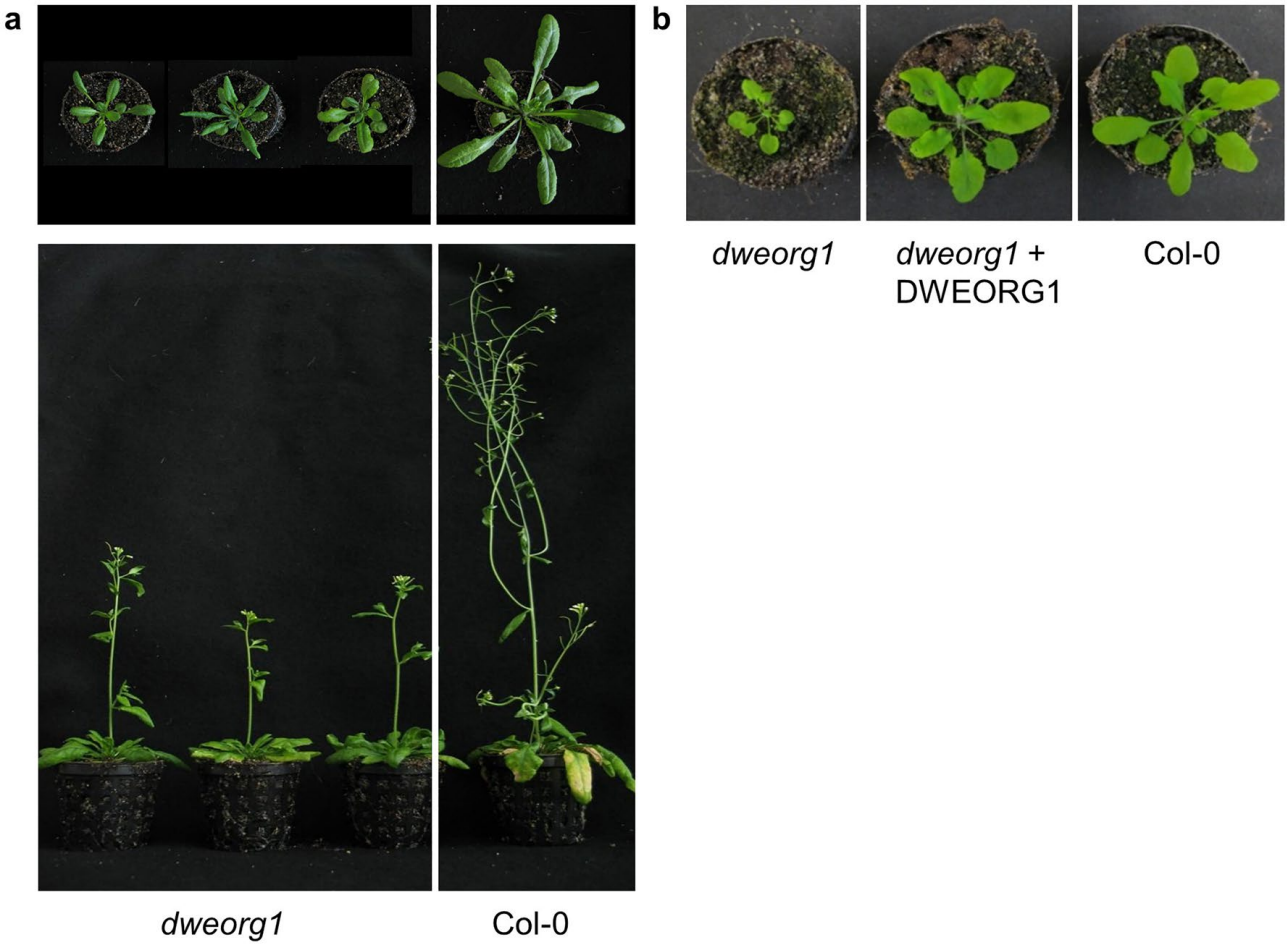
*dweorg1* mutant plants show a slightly reduced size compared with the wild type. (**a**) Growth comparison of wild type (Col-0) and *dweorg1* mutant plants six weeks after sowing under long-day conditions (16 h light/8 h dark; 64% humidity, 22 °C with a light intensity of 120–122 µmol*m^−2^*s^−1^). (**b**) Growth comparison of wild type (Col-0)*, dweorg1*, and *dweorg1*-complemented with wild type *DWEORG1* under the control of the CaMV 35S promoter four weeks after sowing under long-day conditions (16 h light/8 h dark; 64% humidity, 22 °C with a light intensity of 120–122 µmol*m^−2^*s^−1^).

To further study the role of AT3G15590 in *Arabidopsis* mitochondria, the GABI-Kat line GK_188B06 was analyzed. This line contains a T-DNA insertion in the third exon of *AT3G15590*, 974 bp downstream of its start codon (Fig. 1a). Progenies were tested for a homozygous T-DNA insertion via PCR amplification of the wild type allele and the T-DNA insertion in *AT3G15590*. Plants homozygous for the T-DNA insertion were exclusively used in this study and show a dwarf phenotype compared with the wild type (Fig. 2a). In reference to the reduced size of its mutant, the AT3G15590 was renamed DWEORG1 (which means “dwarf” in old English). In homozygous mutant plants, no full-length mRNA derived from *dweorg1* could be detected via reverse-transcription PCR (Supplementary Fig. S1), strongly suggesting that the used mutant line represents a null mutant. The mutant phenotype was reversed by transforming the mutant plants with a functional copy of the *DWEORG1* gene (Fig. 2b), confirming that the observed phenotype was caused by the absence of a functional *DWEORG1* gene.

### The translational efficiency in *dweorg1* mitochondria is reduced

**Figure 3:**
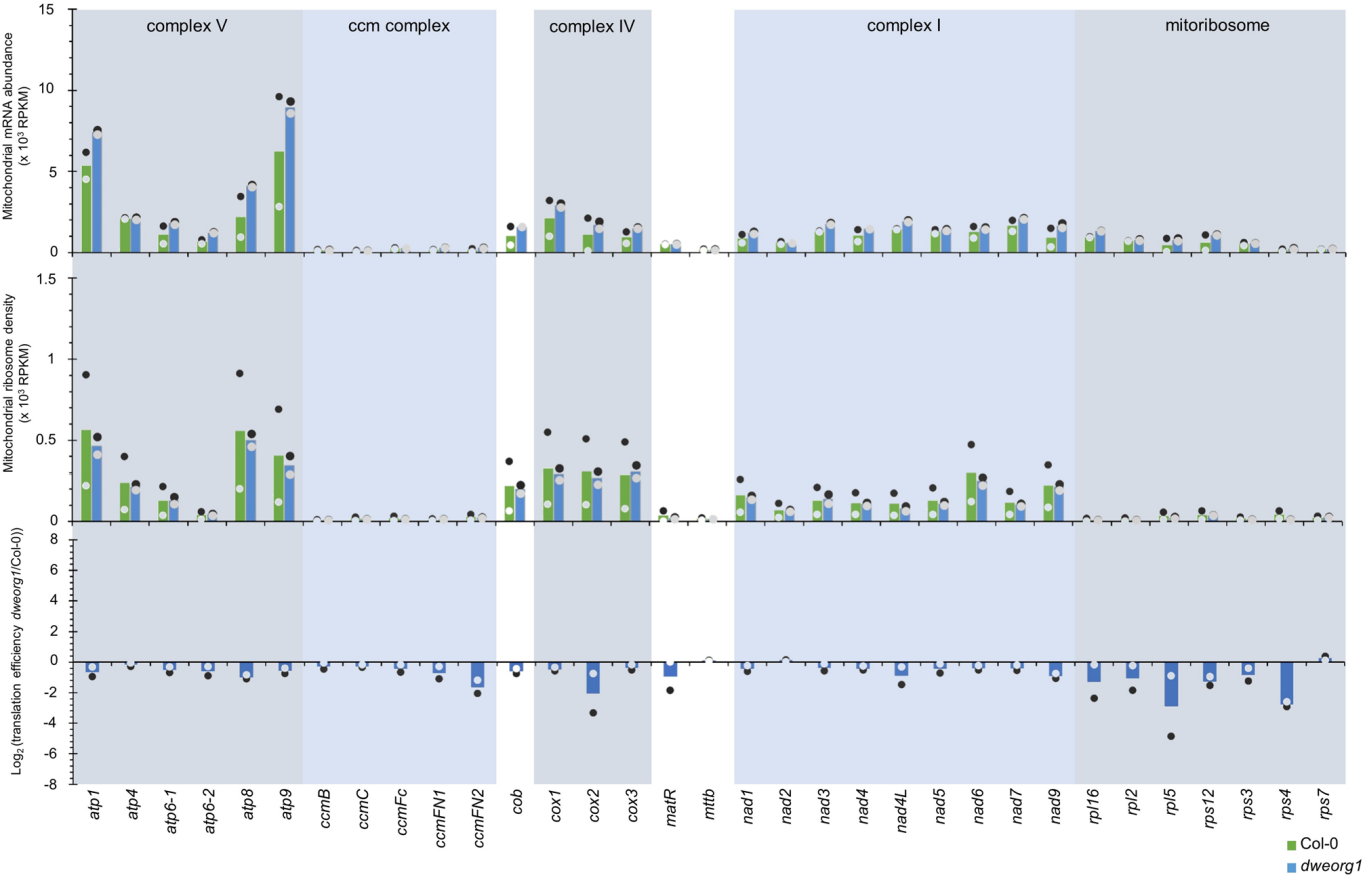
Mitochondrial mRNAs over-accumulate in the *dweorg1* mutant, while the ribosome densities along the mitochondrial mRNAs are reduced. Comparative mitochondrial mRNA levels in wild type and *dweorg1* mutant plants given as RPKM obtained via RNA-seq analysis. Comparison of ribosome densities along mitochondrial mRNAs in wild type and *dweorg1* mutant plants given as RPKM obtained via Ribo-seq. Log_2_ ratios of normalized ribosome footprint abundance in the wild type relative to the *dweorg1* mutant for each mitochondrial mRNA. The values were normalized to the number of Ribo-seq read mappings to the nuclear ORFs, and the abundance of each mRNA was determined via RNA-seq.

rPPR proteins are part of the mitochondrial ribosome in plants, and mutants of these rPPR proteins can show impaired mitochondrial translation^15^. The close similarity between DWEORG1 and rPPR expression patterns led us to investigate the impact of DWEORG1 loss on mitochondrial translation via ribosome profiling analysis^41^. Total ribosome footprints were prepared from flowers of wild type and *dweorg1* mutant plants, mapped to the mitochondrial genome of Col-0 and then quantified in each mitochondrial gene (as previously indicated in Planchard et al.^25^. A statistical analysis of these footprints allows the determination of relative translation levels of the respective mRNAs, thereby allowing a comparison of translational efficiency of all the mitochondria-encoded genes between wild type and *dweorg1* plants. To determine the translational efficiency, the abundance of each mitochondrial mRNA needed to be determined first since it is defined as the average number of ribosome footprints per mRNA^25^. The abundance of mRNAs was determined via RNA-seq using the input sample for the ribosome footprint preparation (before RNase I treatment) and is reported as reads per kilo base per million mapped reads (RPKM) values per mitochondrial gene in Fig. 3. RNA-seq revealed a slight elevation of all mRNAs in *dweorg1* mitochondria except for *atp4*, *ccmB*, *ccmC*, *ccmF*, *matR*, *mttB*, *nad2*, *rpl2*, and *rps7*, which are equal or slightly less abundant in *dweorg1* compared with the wild type (Fig. 3). The normalization of ribosome footprint number by mRNA abundance led us to calculate the translational efficiency for each mitochondrial transcript in wild type and *dweorg1* plants (Fig. 3). A general decrease in ribosome loading per mRNA could be detected for almost all mitochondria-encoded mRNAs in *dweorg1* plants. Most mitochondrial transcripts show a decrease in translational efficiency by a factor of 1.5 to 2 compared with the wild type. However, the translation of several mRNAs such as *cox2*, *rps4*, *rpl5*, and *ccmF*_*N2*_ is more pronounced, with a decrease of up to threefold in translation efficiency of the *rps4* and *rpl5* transcripts (Fig. 3).

### The level of Cox2, Nad9 and Rpl16 is decreased in *dweorg1* plants

**Figure 4:**
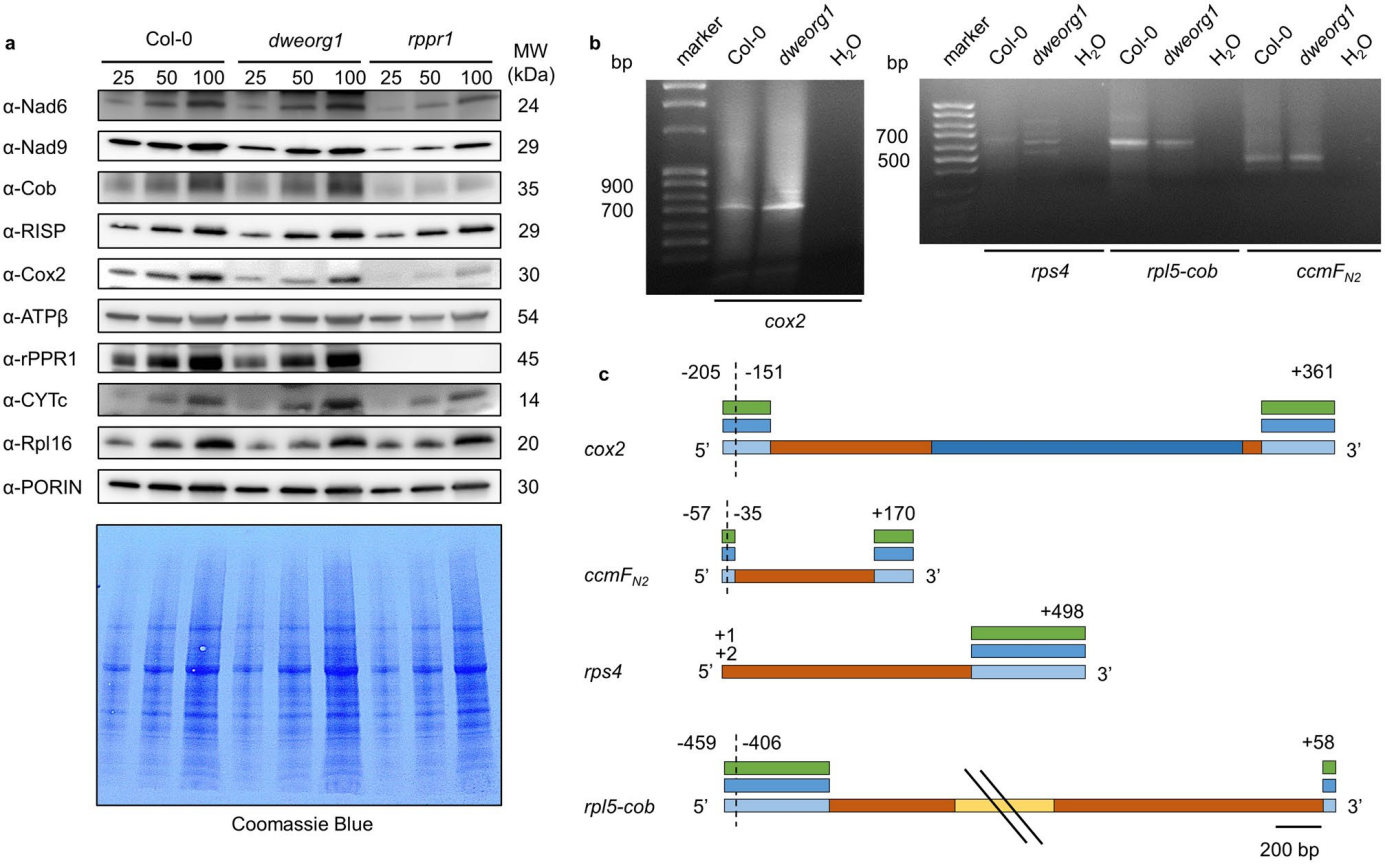
The *dweorg1* mutant accumulates slightly reduced levels of the Cox2 and Nad9 proteins. 5′ and 3′ termini processing for *cox2*, *rps4*, *rpl5*, and *ccmF*_*N2*_ is not affected in *dweorg1*. (**a**) Immunoblot analysis comparing the steady-state levels of selected mitochondria-encoded (Nad9, Cox2, Rpl16, ATPβ) and nuclear-encoded (RISP, rPPR1, CYTc) mitochondrial proteins in wild type, *dweorg1*, and *rppr1* mutants. PORIN was used as a loading control; 25, 50, and 100 µg of total proteins were used in each lane, as indicated. The molecular weight of the respective proteins is given in kDa. Original, full-length blots are presented in Supplementary Fig. S10. (**b**) cRT-PCR analysis amplifying the 5′ and 3′ termini of *cox2*, *rps4*, *rpl5-cob*, and *ccmF*_*N2*_ transcripts. Total RNAs were extracted from the indicated genotypes, circularized, and then used for cDNA synthesis prior to PCR amplification using divergent primers near the mRNA extremities. The obtained PCR amplification products were run on the shown agarose gel. H_2_O was used for PCR water control. Original, full-length gels are presented in Supplementary Fig. S11. (**c**) Illustration of the determined 5′ and 3′ termini of *cox2*, *rps4*, *rpl5-cob*, and *ccmF*_*N2*_in wild type and *dweorg1* mutant plants via cRNA analysis, quantitative RT-PCR, and reverse transcription PCR. Depicted is the structure of *cox2*, *rps4*, *rpl5-cob*, and *ccmF*_*N2*_ mRNA, exons being shown as orange boxes, introns as dark blue boxes, intergenic regions (not to scale) as yellow boxes, and the 5′ and 3′ termini as light blue boxes. The 5′ and 3′ termini are shown as identified in^24^.

As the overall translation in *dweorg1* mitochondria appeared to be negatively affected, the abundance of several mitochondrial proteins was analyzed via western blot analysis. Antibodies against mitochondria-encoded proteins such as Nad6 and Nad9 (complex I), Cob (complex III), Cox2 (complex IV), Rps4 and Rpl16 (mitoribosome), and nuclear-encoded mitochondrial proteins such as RISP (complex III), ATPβ (complex V), rPPR1, and CYTc were used in this analysis. To analyze the western blot results grey values of the protein bands of interest where determined for comparing the protein level in *dweorg1* and wild type plants (Supplementary Fig. S4). This analysis showed, that the Cox2 protein level was significantly reduced in *dweorg1*. In addition the levels of Nad9 and Rpl16 seem to be slightly reduced in *dweorg1* while no significant differences in other protein levels could be detected (Fig. 4a, Supplementary Fig. S3 and S4). In the lines complemented with wild type *DWEORG1*, the level of Cox2 protein was restored to that of wild type (Supplementary Fig. S3). Although the protein levels of Cox2 and Nad9 are decreased in *dweorg1*, BN-PAGE gel analysis did not reveal any visible reduction in complex I and IV accumulation compared with the wild type, as for other respiratory complexes (Supplementary Fig. S5). As a control, the steady-state levels of the same proteins were measured in the *rppr1* mutant and compared with that in *dweorg1*. In the case of *rppr1,* a more drastic decrease in Cox2 protein accumulation as well as all other tested mitochondria-encoded proteins (i.e., Nad6, Nad9 and Cob) could be detected.

### Mitochondrial mRNA processing is not negatively affected in *dweorg1*

To better understand the function of DWEORG1, its potential role in mitochondrial RNA processing was also investigated. As already indicated, the RNA-seq results did not reveal any loss of any mitochondria-encoded mRNA in mutant plants, suggesting that DWEORG1 does not carry any obvious stabilization activity toward certain mitochondrial mRNAs (Fig. 3). The splicing efficiency of each mitochondrial intron was next quantified via RT-quantitative PCR analysis, and indeed, some introns showed an improved splicing efficiency in *dweorg1* plants compared with the wild type, ruling out any negative effect of the missing DWEORG1 on mitochondrial splicing (Supplementary Fig. S6). The editing status of mitochondrial transcripts was also investigated via the sequencing of RT-PCR products of the mitochondrial transcripts, which covered 180 editing sites, but no difference to the wild type was detected (Supplementary Table S2). In addition, a SNaPshot analysis^42–44^ of 408 editing sites revealed no changes in RNA editing in the *dweorg1* mutant compared with the wild type (Supplementary Table S3).

As disturbed mRNA end formation could impact protein production in plant mitochondria^45^, the 5′ and 3′ termini of *cox2*, *rps4*, *rpl5*, and *ccmF*_*N2*_ transcripts were comparatively analyzed via circular RT-PCR (cRT-PCR) analysis in wild type and *dweorg1* plants, since these transcripts’ translational efficiency was most affected in *dweorg1*. No difference in the size of the amplified products could be observed in the wild type and *dweorg1* samples for *ccmF*_*N2*_, *cox2*, and *rpl5* (Fig. 4b). In the case of the *rps4* transcript, different-sized products were detected in *dweorg1* but not in the wild type. The generation of larger PCR products with this method was shown before by Forner et al.^24^. The different 5′ and 3′ termini for the *cox2*, *rps4*, *rpl5*, and *ccmF*_*N2*_ transcripts are shown in Fig. 4c. Mature *cox2* mRNAs bear 5′ termini located either 205 or 151 nucleotides upstream of the start codon and a 3′ terminus positioned 361 nucleotides downstream of the stop codon. Mature *rps4* mRNAs bear a 5′ termini located at position + 2 (+ 1 = the first base of the start codon) and a 3′ terminus positioned 498 nucleotides downstream of the stop codon. The dicistronic mature *rpl5-cob* mRNAs bear 5′ termini located either 459 or 406 nucleotides upstream of the start codon and a 3′ terminus positioned 58 nucleotides downstream of the stop codon. Mature *ccmF*_*N2*_ mRNAs bear 5′ termini located either 57 or 35 nucleotides upstream of the start codon and a 3′ terminus positioned 170 nucleotides downstream of the stop codon^24^. The sequencing of cRT-PCR amplification products showed that the major form of *cox2* (− 151/+ 361) and *ccmF*_*N2*_ (− 35/+ 170) accumulate normally in *dweorg1* and wild type plants (Fig. 4c). For *rpl5-cob* transcripts, sequencing confirmed a − 405/+ 58 form in both wild type and *dweorg1* plants. The sequencing results for *rps4* detected a + 2/+ 202 form in the wild type but a + 1/+ 498 form in *dweorg1*. Since the codon UUG, 39 nt downstream of the annotated *rps4 s*tart codon is likely used as the translation initiation start codon, this difference should have no effect on the translation of *rps4* in *dweorg1*^46^.

To detect and quantify the − 205 5′ terminus of *cox2*, quantitative RT-PCR was employed using a forward PCR primer starting 205 nucleotides upstream of the *cox2* start codon (Supplementary Fig. S7). The relative expression value in *dweorg1* showed no significant difference with the wild type regarding the 3′ terminus level of *cox2* transcripts of *dweorg1*. For the 5′ terminus, a slight but significant increase was observed in *dweorg1* compared with the wild type. To detect the − 57 5′ terminus of *ccmF*_*N2*_, the − 459 5′ terminus of *rpl5-cob*, and the + 498 3′ terminus of *rps4*, RT-PCR was employed using a PCR primer starting 57 nucleotides and 459 nucleotides upstream of the *ccmF*_*N2*_ and *rpl5-cob* start codons, respectively, as well as a PCR primer ending 498 nucleotides downstream of the *rps4* stop codon. In all three cases, PCR products were obtained representing the expected size for the analyzed termini (Supplementary Fig. S7), indicating intact 5′ and 3′ termini for the analyzed transcripts. Together with the results of the cRT-PCR analysis, these findings suggest that the decrease in translational efficiency of *cox2*, *rps4*, *rpl5*, and *ccmF*_*N2*_ is not caused by modified extremities of corresponding mature mRNAs in *dweorg1* plants.

### DWEORG1 sediments with ribosomes

**Figure 5:**
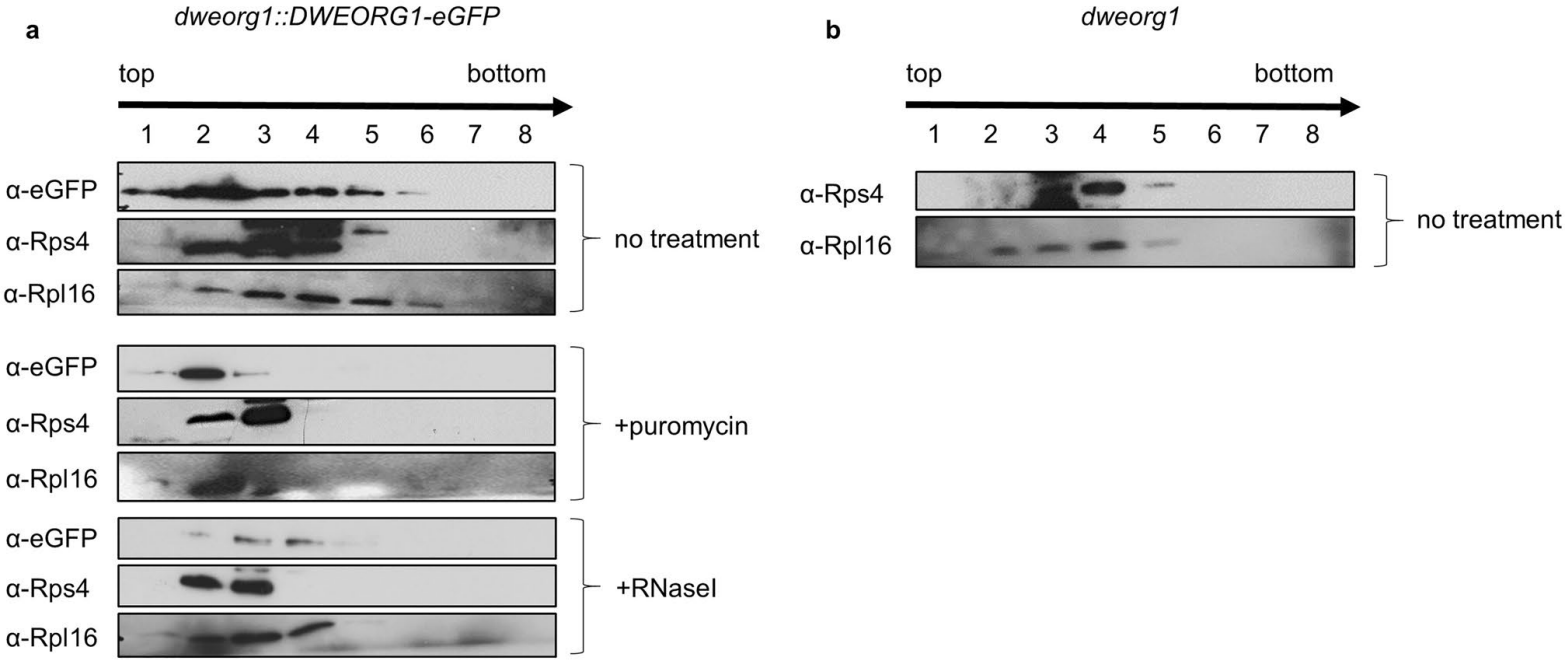
The DWEORG1 protein co-sediments with the *Arabidopsis* mitoribosome in a sucrose gradient. (**a**) Mitochondrial extracts prepared from root cultures of *dweorg1* mutants complemented with a DWEORG1–eGFP construct were fractionated on 15%–55% sucrose gradients. An equal volume of each recovered fraction was analyzed via protein gel blot with the indicated antibodies (α-eGFP, α-Rps4 and α-Rpl16 detect proteins of 100 kDa, 40 kDa and 20 kDa, respectively); 1 refers to the top of the gradient and 8 to the bottom. The mitochondrial extracts were either not treated or treated with puromycin or RNaseI to destabilize the ribosomes. To detect mitoribosomal particles, antibodies against mitochondrial Rps4 and Rpl16 were used. (**b**) Mitochondrial extracts prepared from *dweorg1* root cultures were fractionated on 15%–55% sucrose gradients; 1 refers to the top of the gradient and 8 to the bottom. Mitoribosomal particles were detected with antibodies against mitochondrial Rps4 and Rpl16. Original, full-length gels are presented in Supplementary Fig. S12.

Since the *DWEORG1* gene shows an expression pattern similar to those of *rPPR* genes, an association of DWEORG1 with *Arabidopsis* mitoribosomes was analyzed via sucrose gradient sedimentation analysis. For this analysis *dweorg1* plants expressing a C-terminal fusion of DWEORG1 with eGFP were used. Expression of the DWEORG1-eGFP complemented the *dweorg1* phenotype (Supplementary Fig. S8). To this end, mitochondrial extracts prepared from plants expressing a DWEORG1–eGFP fusion were fractionated on a continuous 15%–55% sucrose gradient, and the eight recovered fractions were analyzed using immunoblot assays. To localize mitoribosomes within the gradients, antibodies to the mitoribosomal proteins Rps4 and Rpl16 were used, two proteins belonging to the small and large subunits of mitoribosomes, respectively. Rpl16 and Rps4 are both peaking in fractions three and four, respectively (Fig. 5a). DWEORG1 is peaking similarly to Rps4 and Rpl16 in fractions two to four, while being detected in fractions one to six. To confirm an association of DWEORG1 with the mitoribosomes, the analysis was repeated under ribosome-destabilizing conditions by treating the mitochondrial extracts with 0.6 mM puromycin or 5 U RNase I before loading them on the gradients. Puromycin leads to a direct destabilization of the ribosomes, whereas RNase I treatment destabilizes ribosomes by degrading rRNAs^47^. In both cases, a shift of ribosomal proteins toward upper fractions was observed, as for DWEORG1 (Fig. 5a), further supporting an association of DWEORG1 with the mitochondrial ribosomes.

When separating the mitochondria extract isolated from *dweorg1* plants on the sucrose gradient, Rps4 was detected in fractions three to five (peaking in fraction three and four) and Rpl16 in fractions two to five (peaking in fraction four), while the signal in fraction five is very weak (Fig. 5b). This suggests less loading of mitoribosomes on mRNAs in *dweorg1,* contributing to a lower translation efficiency in the mutant, thus confirming the results shown in Fig. 3.

### The accumulation of mitochondrial rRNAs is reduced in *dweorg1* mutants

**Figure 6:**
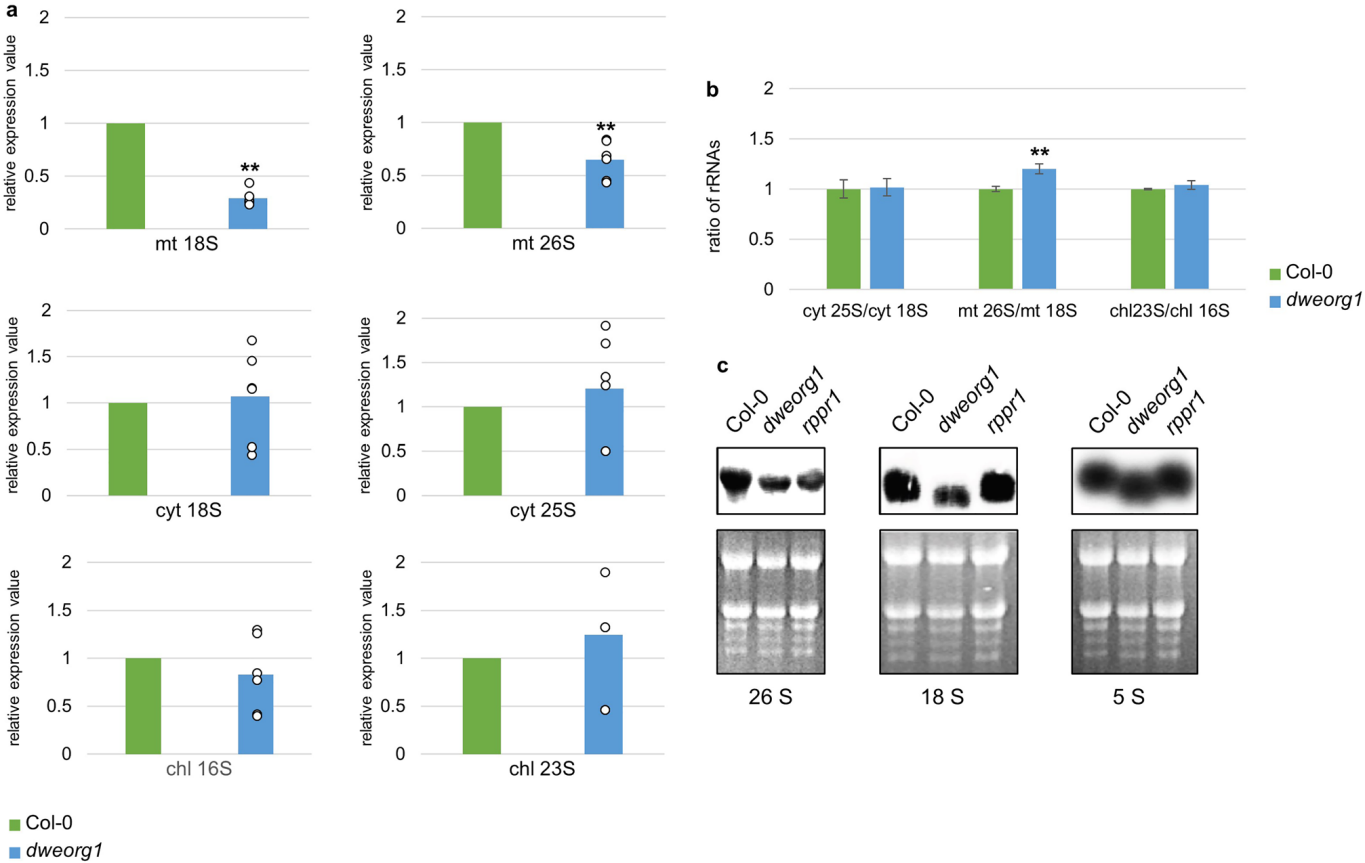
The mitochondrial rRNAs under-accumulate in *dweorg1* plants. (**a**) The *dweorg1* mitochondrial, chloroplast, and cytosolic rRNA levels were determined via quantitative RT-PCR in relation to the wild type. *act2* was used as an internal control. The values shown are the mean of three biological and two technical repeats with ± SDs (**P < 0.01, student’s *t*-test). (**b**) Ratios between rRNA levels of mitochondrial, chloroplastic, and cytosolic LSU and SSU. The values shown are the means of three biological and two technical repeats with ± SDs (*P < 0.05, **P < 0.01, student’s *t*-test). (**c**) RNA gel blot analysis of wild type, *dweorg1*, and *rppr1* mitochondrial ribosomal rRNAs. The total RNA was extracted from the indicated genotypes, separated on an agarose gel, and blotted onto a nylon membrane, and the membrane was hybridized with probes to the mitochondrial 26S, 18S, and 5S rRNAs. Original, full-length gels are presented in Supplementary Fig. S13.

The rRNA level can be used as a proxy for ribosomal subunit accumulation since rRNAs are unstable when unassembled^48^. The levels of cytosolic 18S and 25S rRNAs, chloroplast 16S and 23S rRNAs, and mitochondrial 18S and 26S rRNAs were determined via quantitative RT-PCR analysis. As seen in Fig. 6a, no difference in rRNA levels could be detected for both cytosolic and chloroplast rRNAs; however, a significant reduction in mitochondrial rRNAs was observed in *dweorg1* plants compared with the wild type. The mitochondrial 18S rRNA in *dweorg1* was reduced to 30% of the wild type levels, while the mitochondrial 26S rRNA was only reduced to 65% of the wild type levels (Fig. 6a). Based on the respective rRNA level, the ratios among the cytosolic, chloroplast, and mitochondrial rRNAs of the LSU and SSU rRNAs were calculated (Fig. 6b). While the LSU/SSU ratio in the cytosol and chloroplast showed no significant difference in the wild type, a significant increase of this ratio was found in *dweorg1*, confirming an imbalance of SSU and LSU in *dweorg1* mitochondria, while the cytosolic and chloroplast ribosomes are not affected. The decrease in 18S and 26S rRNA in the *dweorg1* mutant was confirmed via Northern blot analysis (Fig. 6c).

## Discussion

### DWEORG1 is likely a novel mitochondrial rPPR protein

PPR proteins constitute a large family of plant proteins facilitating organellar RNA expression through processes such as RNA editing, splicing, 5′- and 3′-end maturation, RNA stability, and translation^5–7^. Additionally, PPR proteins were recently reported to be part of the plant mitoribosome. So far, 11 ribosomal PPR (rPPR) proteins were identified—rPPR1, rPPR2, rPPR3a, rPPR3b, and rPPR4-10^15,16,36^. Therefore, PPR proteins can affect the translation of mitochondrial proteins either directly or indirectly, depending on the process they are involved in. The evidence provided in this study suggests a direct role of DWEORG1 in mitochondrial translation through an association with the mitoribosome as a novel rPPR protein.

Most mutations in a PPR protein lead to a severe phenotype of the plant since they affect vital biological processes such as plant growth, development, and reproduction^5,6^. This is not true for the *dweorg1* mutant; here, we only observe a mild growth delay (Fig. 2), just like rPPR mutants *rppr1* or *rppr3a*, *rppr3b*, and *rppr8*, which also show a mild growth delay or do not show any macroscopic phenotype at all, respectively^15^. Another feature of DWEORG1 is its high expression during all developmental stages compared with other P-type PPR proteins with a much lower expression (Fig. 1e). The same expression pattern is seen for rPPR proteins (Fig. 1d). Furthermore, DWEORG1’s copy number of 926 copies/mitochondrion is comparably high for a PPR protein; most other PPR proteins are only found at 1 and 20 copies/mitochondrion, while rPPR proteins are found at copy numbers similar to DWEORG1^37^. All this makes DWEORG1 a good candidate for a novel rPPR protein. One of the identified rPPR proteins, rPPR1, was originally shown to be associated with polysomes and named PPR336^49^. When it was later found to be a core protein of the mitoribosome, it was renamed rPPR1, and an overall decrease in translational efficiency of mitochondrial transcripts was shown in *rppr1* mutants^15^. Indeed, we could show that DWEORG1 does have a similar effect on mitochondrial translation as rPPR1 but to a lesser degree. We could show an overall decrease of the translational efficiency of mitochondrial transcripts, with *cox2*, *rps4*, *rpl5*, and *ccmF*_*N2*_ transcripts being the most affected (Fig. 3). While the translational deficiency in *rppr1* is reflected at the mitochondrial protein level, it is different in *dweorg1*. For *cox2* the decrease in translational efficiency could also be detected at the protein level, as well as for *nad9* and *rpl16* but not for *rps4* or the other analyzed mitochondria encoded proteins (Fig. 4 and Supplementary Fig. S3). This might be due to the minor decrease in translational efficiency for most of the mitochondrial transcripts requiring a more sensitive detection method than a western blot. Another reason for this could be a compensation of the decreased translation via a decreased protein degradation rate in *dweorg1*, especially in the case of important conserved proteins like Rps4. This has yet to be further analyzed.

The translation efficiency of mitochondrial transcripts can be affected by PPR proteins directly as some are part of the mitoribosomes and others act as transcript-specific translational activators or indirectly because of the defective RNA processing of specific transcripts. The data presented here points in the direction of a direct role of DWEORG1 by interacting with the mitoribosomes. If DWEORG1 acts as a transcript-specific translational activator (directly or indirectly) for *cox2*, *rps4*, *rpl5*, and *ccmF*_*N2*_, the overall decrease in the translational efficiency of the other mitochondrial transcripts could be a secondary effect. Up to now, very few proteins have been found to control translation in plant mitochondria, all belonging to the PPR protein family. RF1 and PPR-B act as restorers of fertility in rice (*Oryza sativa*) and radish (*Raphanus sativus*), respectively, by negatively regulating the translation of cytoplasmic male sterility (CMS), causing transcripts *orf138* in radish and *orf79* in rice, respectively^50–52^. MPPR6 interacts with the 5′-UTR of *rps3* mRNA in maize, and *mppr6* mutants express 5′-extended *rps3* transcripts whose translation is likely impaired, causing an overall reduction of mitochondrial translation^53^. MTL1, an *Arabidopsis* PPR protein, is directly required for translation initiation of *nad7* mRNA. MTL1 does not affect the 5′-processing of *nad7* mRNA just as Rf1 and MPPR6 do with their target RNA but rather directly controls *nad7* translation through a mechanism that still needs to be understood^54^. In contrast to chloroplasts, mitochondrial transcripts do not possess a Shine-Dalgarno sequence or any other obvious ribosome binding site^21,22^. It is therefore believed that gene-specific *trans*-factors such as PPR proteins guide the ribosomes to the site of translation initiation. Binding to the mRNA’s 5′-UTR could be one way of action for these *trans*-factors by altering its structure and thereby making it accessible for the mitoribosomes. This has been proposed for PPR10 in maize chloroplasts, the only PPR protein for which the molecular mode of action in translation initiation has been further analyzed. PPR10 binds to the 5′-UTR of *atpH* mRNA, preventing the formation of a stem-loop structure and thereby making the Shine-Dalgarno sequence accessible for the small ribosomal subunit^55^. A function similar to PPR10 seem unlikely for DWEORG1. DWEORG1 does not follow the postulated PPR-code (Supplementary Fig. S9), hence no putative RNA binding site of DWEORG1 could be predicted. In addition, no sequence similarities are detected in the 5′ ends of *cox2*, *rps4*, *rpl5*, and *ccmF*_*N2*_ that could act as target sequences for DWEORG1. Hence, a sequence-specific translation activation through DWEORG1 is unlikely for these transcripts. In addition, no defect in RNA processing for any mitochondrial transcript was detected; all transcripts are transcribed and stable, show intact 5′- and 3′-UTRs, are edited correctly, and are normally spliced (see Fig. 4, Supplementary Fig. S4, S5, Supplementary Table S2 and S3).

These findings, in addition to the similarities to *rppr1* mutants, lead to the conclusion that DWEORG1 could be a non-previously reported rPPR protein and also be part of the plant mitoribosome. This is supported by our finding that DWEORG1 containing particles fractionate with ribosomes (Fig. 5) as well as by the fact that in a study of plant mitoribosomes, DWEORG1 was also found to be co-sedimenting in sucrose gradients with mitoribosomal components^16^. Since DWEORG1 was co-precipitated with ribosomal components under stabilizing conditions but not under destabilizing conditions^15^, it seems more likely that DWEORG1 functions as a variable part of mitoribosomes rather than as a core protein of mitoribosomes. Defects in mitoribosomal proteins can have different effects on the mitochondrial translation. While *rppr1* mutants show a more or less equal decrease in translational efficiency for all mitochondrial transcripts^15^, silencing the *RPS10* gene in *Arabidopsis* leads to an increase in the translation of mRNAs coding for mitoribosomal proteins and a decrease in the translation of OXPHOS components^27^. In the case of *dweorg1* mutants, the translational efficiency of all mitochondrial transcripts is slightly decreased, with four transcripts (*cox2*, *rps4*, *rpl5*, and *ccmF*_*N2*_) being more affected than the rest of the mitochondrial transcripts. These transcripts cannot be assigned to just one category since they are coding for mitoribosomal proteins as well as for an OXPHOS component and a component of the cytochrome c biogenesis. Why these four transcripts are more affected has yet to be further analyzed. Rps4 and Rpl5 are both conserved r-proteins^15,16^, a deficiency in their translational rate could cause the overall decrease in mitochondrial translation efficiency as seen for a *rps3* mutation on maize plants showing an overall disturbed proteinsynthesis^53^. But as shown in *rps10* plants, a low r-protein level usually leads to a general increase of translational rates of mitoribosomal proteins, which is not seen in *dweorg1*. Hence it is more likely that the decrease in translational efficiency seen in *dweorg1* is a direct effect of the loss of DWEORG1.

### DWEORG1 may have an rRNA-stabilizing function

Organellar ribosomes originate from prokaryotic ribosomes. While ribosomes in chloroplasts strongly resemble bacterial ribosomes, mitoribosomes diverged strongly in their structure, rRNA, and protein content from the ancestral ribosomes during eukaryotic evolution^56,57^. Mitoribosomes are composed of two ribonucleoprotein complexes: the LSU and the SSU. These complexes show a distinct combination of rRNAs and proteins that is distinct for different eukaryotes^58^. The plant mitoribosome, with its many rRNA ESs and plant-specific ribosomal proteins (r-proteins), is one of the most complex mitoribosomes^15^. One distinct feature of plant mitoribosomes is their 11 rPPR proteins, a feature only shared by mammalian and trypanosomal mitoribosomes, containing six and two rPPRs, respectively^15,16,59,60^. In animals, the rPPR protein mS39 is found to bind to leaderless mitochondrial mRNAs, leading them to the entrance channel of the ribosome^61^. While some rPPRs in trypanosomal mitoribosomes are only present as structural components of the ribosome and do not interact with the RNA at all^62^, other rPPRs in trypanosomes assemble around the shortened rRNAs, stabilize and position their functionally important regions in the ribosome^60^. A stabilizing function for rRNAs is also seen for plant rPPRs; the foot and head extensions of the plant mitoribosome SSU are stabilized by rPPR1, rPPR3a, or rPPR3b and rPPR6, respectively^36^. The same is seen for the extension segments of the 26S rRNA of the LSU.

Here, we have shown a decrease in the 26S and 18S rRNA levels in the *dweorg1* mutant (Fig. 6), suggesting a role for DWEORG1 in rRNA stabilization. Reduced rRNA levels are a good indicator of ribosome assembly since non-assembled rRNAs are very unstable^48^. In *dweorg1* mutants, we could observe a shift of ribosomal proteins Rps4 and Rpl16 to the upper fractions of a sucrose gradient (Fig. 5b). The upper fractions of the sucrose gradient represent monosomes attached to RNA, free and unassembled ribosomal subunits. This is in accordance with the decrease in translational efficiency in the mitochondria of *dweorg1* plants and hints at a disturbed mitoribosome biogenesis in the mutant compared with the wild type.

A defect in mitoribosomal biogenesis is also seen in mitochondrial *rps10* mutants^27^. The translation of mitochondrial transcripts is maintained in the *rps10* mutant as well as in the *dweorg1* mutant despite the disturbed mitoribosome biogenesis. In both cases, a change in translational efficiency of all mitochondrial transcripts can be observed, but the decrease or increase affects the various transcripts differently in the two mutants. Since two of the transcripts affected by DWEORG1 encode for ribosomal proteins—namely, Rps4 and Rpl5—there are two possibilities for the unstable ribosomes: (i) DWEORG1 itself stabilizes rRNAs and thereby mitoribosomes in an, up to now, unknown way; or (ii) the destabilization of the mitoribosomes is a secondary effect caused by the insufficiently translated mitoribosomal proteins Rps4 and Rpl5, leading to incompletely assembled/unstable mitoribosomes. In the latter case, one would expect an increase in the translation of other mitoribosomal protein mRNAs, as seen in the *rps10* mutant^27^, but this is not the case in *dweorg1* mitochondria. Thus, excluding scenario (ii). The data received in this study shows an imbalance of SSU and LSU in mitochondria, with an excess of LSU compared with SSU (Fig. 6b). Although the molecular details remain to be determined, our data indicate a stabilizing role of DWEORG1 for the small subunit 18S rRNA of mitoribosomes in *A. thaliana* in addition to an up to now unknown promoting effect on the translation of *cox2*, *rps4*, *rpl5*, and *ccmF*_*N2*_.

In plants, the hypothesis of a heterogeneity of mitoribosomes exists and could allow a highly dynamic mitochondrial translation machinery composition^63^. Studies on LSU proteins in yeast showed that many r-proteins, conserved and specific, are not essential for functional mitoribosomes^64^, suggesting the possibility for differentially composed mitoribosomes. Existing evidence shows a different composition of mitoribosomes in different developmental stages and/or tissues in *Arabidopsis*. The mRNA levels of HUELLENLOS (HLL) and HUELLENLOS PARALOG (HLP), two paralogs of the mitoribosomal protein RPL14, differ in carpels and leaves. HLL is significantly more expressed in carpels than HLP, and the opposite is seen in leaves^65^. Since the two proteins are redundant in their function when expressed ectopically, it can be assumed that they are integrated into mitoribosomes in a tissue-specific manner. A changing mRNA level during leaf development is also observed for many other nuclear-encoded mitoribosomal proteins^63^. RPL14 is not the only mitoribosomal protein with different paralogues in *Arabidopsis*; for RPL12, four paralogues that are all part of the mitoribosome were described^66^. This could reflect a heterogeneous population of mitoribosomes, supporting the hypothesis of heterogeneity in mitoribosomes. A differential composition of mitoribosomes could allow for a regulative role of mitoribosomes in gene expression. DWEORG1 might also have a regulative role as part of the mitoribosome. The expression pattern of *DWEORG1* resembles that of rPPR protein genes in all developmental stages, except the stem elongation phase (Fig. 1). In this phase, the expression of *DWEORG1* is higher, than that of other rPPR genes, indicating a possible role of DWEORG1 during stem elongation as part of the mitoribosome. This could also explain the mild effect on translational efficiency seen here, since the data for translational efficiency in *dweorg1* were collected from inflorescences of Arabidopsis that form after the stem elongation phase.

The ribosomal filter hypothesis postulates that ribosomes are not only the passive site for translation but also able to regulate translation and therefore gene expression by choosing specific mRNAs for translation as a response to different physiological conditions as well as different developmental stages^67–69^. This was shown for yeast mitoribosomes; the r-protein mS38 is specifically needed for the translation of *cox1*, *cox2*, and *cox3* transcripts^70^. In *mS38* mutants, a reduction of the overall translation rate of mitochondrial transcripts is observed, but still, all mitochondrially encoded proteins were synthesized in normal amounts except for the Cox1, Cox2, and Cox3 proteins, which are synthesized in a reduced amount. This seems to be similar in *dweorg1* mutants (Fig. 4a). One process in plant mitochondria that is already proven to be regulated by translation is OXPHOS biogenesis^71^. This feature might enable plant mitoribosomes to translate transcripts without a distinct ribosome-binding site and to target specific mRNAs under different conditions. This theory would also explain why mitoribosomes can still translate mitochondrial transcripts when ribosomal proteins are not synthesized in a sufficient amount. If the heterogeneity of mitoribosomes exists, the composition of the ribosomes is variable to a certain degree, allowing for functional ribosomes even when one ribosomal protein is missing. The findings of this study support this theory.

## Methods

### Plant materials

The *A. thaliana* ecotype Columbia-0 (Col-0) was used as the wild type reference. The GK-188B06 T-DNA insertion line (*dweorg1*) was obtained from the European Arabidopsis Stock Centre (NASC). *A. thaliana* plants were grown under controlled conditions in a climate chamber (22 °C, 60% humidity, 8 h light and 16 h dark) or a greenhouse (8 h light and 16 h dark). Plants were grown on soil or under sterile conditions on Murashige and Skoog (MS) plates^72^ after surface sterilization of the seeds according to Weigel and Glazebrook^73^. *Arabidopsis* root cultures were obtained by placing one-to-two-week-old seedlings grown on MS media in flasks containing 200 ml of an *Arabidopsis* root culture (ARC) liquid medium (20–30 seedlings/200 ml)^74^. The flasks containing the seedlings were cultured, shaking at 80 rpm in the dark at 25 °C, and after two weeks, 50 µg/L of indole-3-acetic-acid was added to the medium to support root growth. After one more week of culture, the medium was renewed. One to two weeks after this renewal, the cultures were harvested and used for the isolation of mitochondria.

### Complementation by stable transformation

Homozygous *dweorg1* plants were transformed with the *dweorg1* coding sequence under the control of the 35S promoter in the pB2GW7 vector^75^. The DWEORG1–eGFP fusion was created by subcloning the *dweorg1* coding sequence downstream of the *egfp* open reading frame and under the control of the 35S promoter in the pOL-GFPS65C vector^76^. The *Agrobacterium tumefaciens*–mediated genetic transformation of *A. thaliana* was done using the floral dip method^77^. Transgenic-complemented plants were germinated on soil and selected by spraying with 0.1% BASTA herbicide (Hoechst).

### Subcellular localization of DWEORG1

Protoplasts were prepared from ten-day-old dark grown *A. thaliana* suspension cultures MM1^78^. The protoplasts were transformed with the DWEORG1–eGFP fusion construct using the polyethylene glycol (PEG) method^79,80^. After overnight incubation, the mitochondria of the protoplasts were stained with MitoTracker® Orange CM-H2TMRos (Invitrogen) at a final concentration of 500 nM and examined using a Leica confocal laser scanning microscope (Leica Microsystems).

### Isolation of mitochondria and sucrose gradient analysis

The mitochondria of the *Arabidopsis* root cultures were isolated using a procedure previously described in Klein et al.^81^ except that the Percoll gradients were replaced by sucrose gradients^82,83^.

For sucrose gradient sedimentation analysis, 3 mg of mitochondria were first lysed in a lysis buffer (0.2 M Tris–HCl (pH 9.0), 0.2 M KCl, 35 mM MgCl_2_, 25 mM EGTA, 0.2 M sucrose, 1% (v/v) Triton X-100, 2% polyoxyethylene-10-tridecylether, 1 mg/ml heparin, 0.1 M β-mercaptoethanol, 100 µg/ml chloramphenicol, and 25 µg/ml cycloheximide). A total of 800 µl of the obtained supernatant was layered onto an 11 ml 15%–55% sucrose gradient and centrifuged at 200,000 g for 120 min. Eight fractions of 1,400 µl were then manually collected and the proteins from each fraction extracted as described in Mašek et al.^84^. The obtained proteins were further analyzed via immunoblotting analysis.

### RNA analysis

Total RNA was extracted from flower buds using the TRIzol reagent (Thermo Fisher Scientific), following the manufacturer’s instructions. To remove genomic DNA contamination from total RNA preparations, the DNase Max Kit (Qiagen) was used. For quantitative RT-PCR analysis, either 35 ng of total RNA was reverse-transcribed and amplified in a single tube with a volume of 15 µl using the QuantiTect SYBR Green RT-PCR Kit (Qiagen) or reverse transcription was performed separately on 1 μg of the total RNAs using a random hexamer. Quantitative RT-PCR for analyzing splicing efficiency in plant mitochondria was carried out, as previously described in Haili et al.^54^, using the described set of primers. Two biological repeats and three technical repeats were performed on each genotype. cRT-PCR was performed as described in Forner et al.^24^. RNA gel blot analysis was performed as previously indicated in Haili et al.^54^. Shortly, 10 µg of total RNA was separated on a 1.5% (w/v) agarose gel. After electrophoresis, the RNAs were transferred to nylon membranes and cross-linked in a UV Stratalinker. Hybridization was performed with specific DNA probes amplified by polymerase chain reaction (PCR) and radiolabeled by the Prime A Gene labeling kit (Promega), following the manufacturer’s instructions.

### Protein analysis

Crude mitochondrial pellets were prepared from plant flower buds as previously described in Dahan et al.^85^. Blue native gel (BN-PAGE) and respiratory complex in-gel activity assays were performed as follows. The total proteins were extracted from crude mitochondrial pellets by lysis in 25 mM Tris–HCl pH 7.5, 75 mM NaCl, 0.5 mM EDTA, 0.5% [v/v] Triton X-100, 0.05% [v/v] SDS, 0.25% Na-deoxycholate, AEBSF 1 mM, cOmplete™, Mini protease inhibitor cocktails (Roche). Protein concentrations were determined with the Bradford protein assay. The separation of total proteins was carried out on 4%–20% SDS-PAGE polyacrylamide gels and then electro-transferred to the PVDF membrane. Protein immunodetection for the BN-PAGE or SDS-PAGE gels was performed according to standard procedure using primary antibodies recognizing PORIN (1:200 dilution; a gift from D. Day, University of Western Australia), Nad9 (1:2,000 dilution^86^), Cox2 (1:1,000 dilution; Agrisera, AS04-053A), ATPβ (1:4,000 dilution; Agrisera, AS05-085), RISP (1:5,000 dilution^87^), CYTc (1:5,000 dilution; Bioscience), rPPR1 (1:2,000 dilution), Nad6 (1:1,000 dilution; Agrisera, AS15-2926), Rpl16 (1:1,000 dilution; Agrisera, AS15-3069), Rps4 (1:1,000 dilution; Agrisera, AS15-3068) and Cob (1:5,000 dilution; Meyer et al., 2018). Secondary goat anti-mouse or anti-rabbit horseradish peroxidase-conjugated antibodies (Sigma) were used. The protein signals were revealed using a chemiluminescence detection kit (Bio-Rad).

### Ribo-seq analysis

The total ribosomal footprints were obtained from a mix of flower buds and open flowers of Col-0 and *dweorg1* plants grown in the greenhouse, as described in Planchard et al.^25^. The total RNA and ribosome footprints were depleted of rRNAs using the RiboMinus™ Plant Kit for RNA-Seq (invitrogen), following the manufacturer’s instructions. The sequencing libraries of total RNA and ribosomal footprints of two biological repeats were prepared using the NEXTflex Rapid Directional RNA-Seq Kit (Bioo Scientific) and the NEXTflex Small RNA-Seq Kit v3 (Bioo Scientific), respectively. Next-generation sequencing was performed by the sequencing facility of the Institut de Biologie Intégrative de la Cellule (Gif-sur-Yvette, France) using Illumina NextSeq technology (single end, 75 nt). Bioinformatic analysis was performed as described in Planchard et al.^25^.

All experimental research did fully comply with the relevant institutional, national, and international guidelines and legislation.

## Supplementary Information

Supplementary Information.

## Data Availability

RNA-Seq data are available in the ArrayExpress database (http://www.ebi.ac.uk/arrayexpress) under accession number E-MTAB-11928. Ribo-Seq data are available in the ArrayExpress database (http://www.ebi.ac.uk/arrayexpress) under accession number E-MTAB-11950. All other datasets generated during and/or analyzed during the current study are available from the corresponding author on reasonable request.
