# Supplementary material for: The P-type pentatricopeptide repeat protein DWEORG1 is a non-previously reported rPPR protein of Arabidopsis mitochondria
Source: Sci Rep. 2022 Jul 21;12:12492. doi: 10.1038/s41598-022-16812-0 (PMC9304396; doi:10.1038/s41598-022-16812-0)
Supplement: Supplementary file 1 — Supplementary Information. [file 41598_2022_16812_MOESM1_ESM.pdf]

Supplementary Information

The P-type pentatricopeptide repeat protein DWEORG1 is a non-previously reported rPPR protein of Arabidopsis mitochondria

Stefanie Grüttner[1,3], Tan-Trung Nguyen[2,3], Anika Bruhs[1], Hakim Mireau[2,*] and Frank Kempken[1,*]

[1]Abteilung Botanische Genetik und Molekularbiologie, Botanisches Institut und Botanischer Garten, Christian-Albrechts-Universität zu Kiel, Olshausenstraße 40, 24098 Kiel, Germany

[2]Université Paris-Saclay, INRAE, AgroParisTech, Institut Jean-Pierre Bourgin (IJPB), 78000, Versailles, France.

[3]Co-first author

Supplementary Figures

Supplementary Figure 1. AlphaFold prediction of DWEORG1

Supplementary Figure 2. Full-length DWEORG1 transcripts are undetectable in *dweorg1* plants

Supplementary Figure 3. Western blot of *dweorg1* plants complemented with wild type DWEORG1 and wild type plants with Cox2 antibody. Western blot of *dweorg1* and wild type with Rps4 antibody

Supplementary Figure 4. Grey values of western blot figures shown in Figure 4a

Supplementary Figure 5. Respiratory chain complexes show no obvious decrease in dweorg1 plants

Supplementary Figure 6. The *dweorg1* plants show no remarkable defects in mitochondrial intron splicing

Supplementary Figure 7. Analysis of 5' and 3' termini of mitochondrial transcripts in *dweorg1* and wild type plants

Supplementary Figure 8. Expression of a DWEORG1-eGFP fusion protein complements the dweorg1 phenotype

Supplementary Figure 9. DWEORG1 does not follow the PPR-code

Full-length blots/gels for cropped images

Supplemantary Figure 10. Full-length blots of western blot figures shown in Figure 4a

Supplemantary Figure 11. Full-length gels of DNA-gels shown in Figure 4b

Supplemantary Figure 12. Full-length blots of western blot figures shown in Figure 5a and b

Supplemantary Figure 13. Full-length blots of northern blot figures shown in Figure 6c

Supplementary Tables

Supplemantary Table S1. Oligonucleotides used in this work

Supplemantary Table S2. Editing status of mitochondrial transcripts analyzed by RT-PCR

Supplemantary Table S3. Summary of SNaPshot-analysis results for the editing status of *dweorg1* mitochondrial transcripts

Supplementary Figures

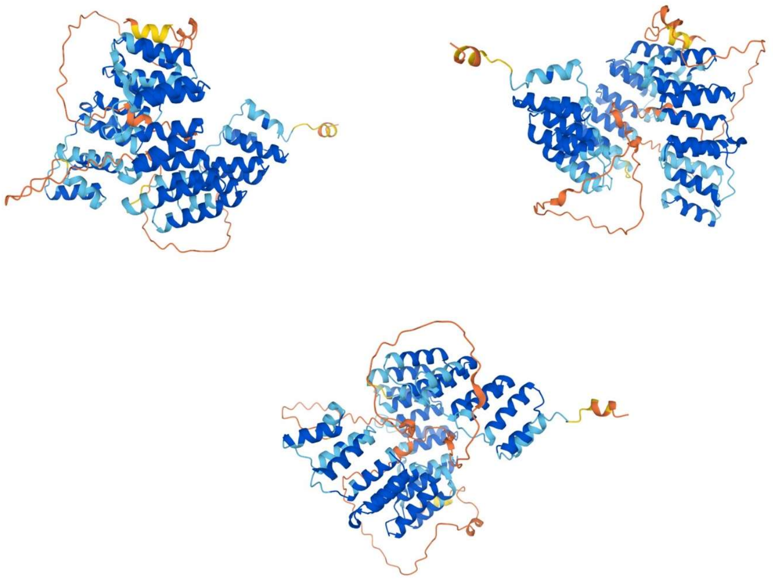

Supplementary Figure 1. AlphaFold prediction of DWEORG1. Structure prediction of DWEORG1 shows repeats consisting of pairs of antiparallel α-helices characteristic for PPR proteins.

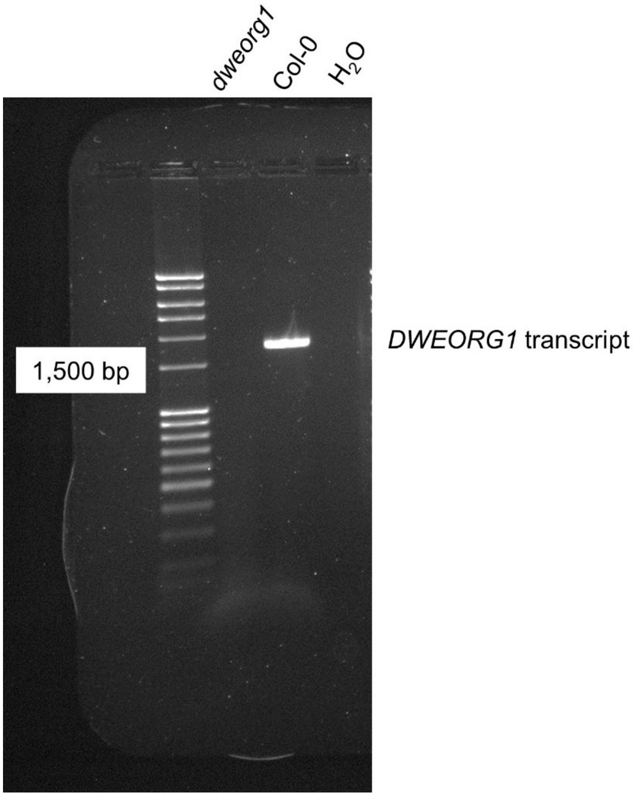

Supplementary Figure 2. Full-length *DWEORG1* transcripts are undetectable in *dweorg1* plants. RT-PCR amplification of the full-length *DWEORG1* cDNA in *dweorg1* and wild type plants.

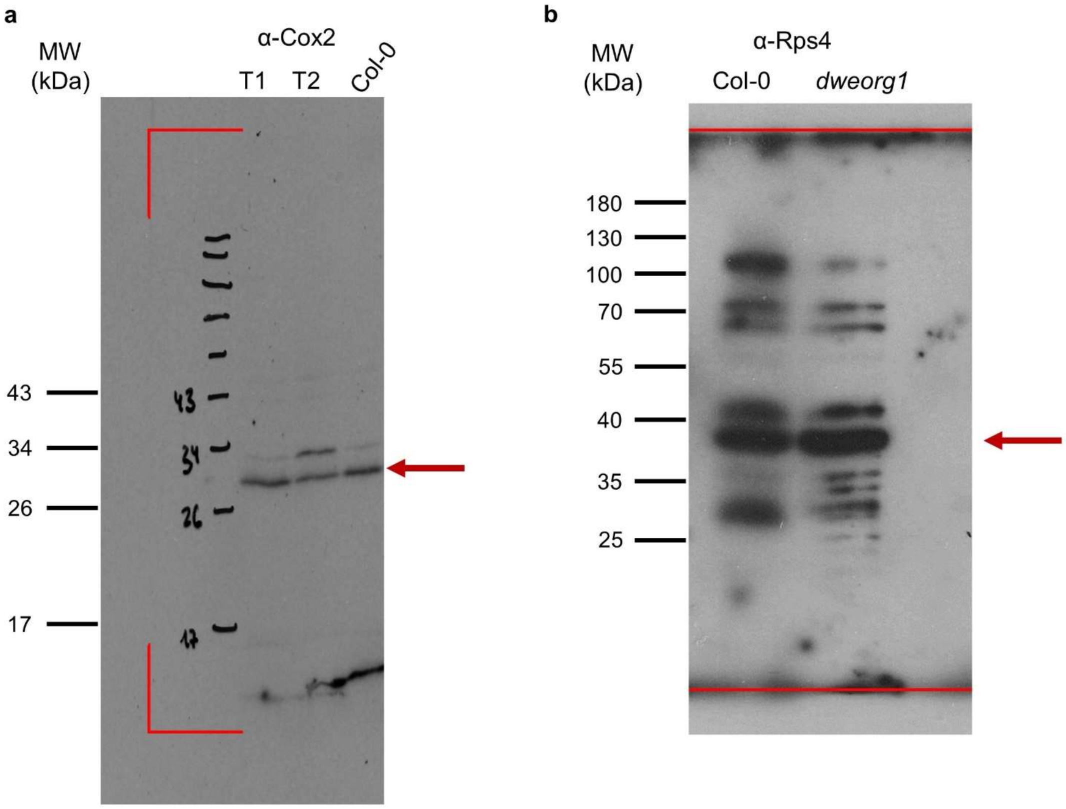

Supplementary Figure 3. *dweorg1* plants complemented with wild type *DWEORG1* show wild type-like steady-state levels of Cox2. *dweorg1* shows wild type-like steady-state levels of Rps4. Membranes were cut prior to hybridization with antibodies. Since the membrane edges are not always visible on the x-ray films red lines indicate the edges. **(a)** Immunoblot analysis comparing the steady-state levels of Cox2 in wild type and *dweorg1* plants complemented with wild type *DWEORG1* (T1 and T2). 30 µg of total proteins were used in each lane, as indicated. (**b**) Immunoblot analysis comparing the steady-state levels of Rps4 in wild type and *dweorg1* plants. 30 µg of total proteins were used in each lane, as indicated. The molecular weight of the respective proteins is given in kDa. Red arrows indicate protein band of interest.

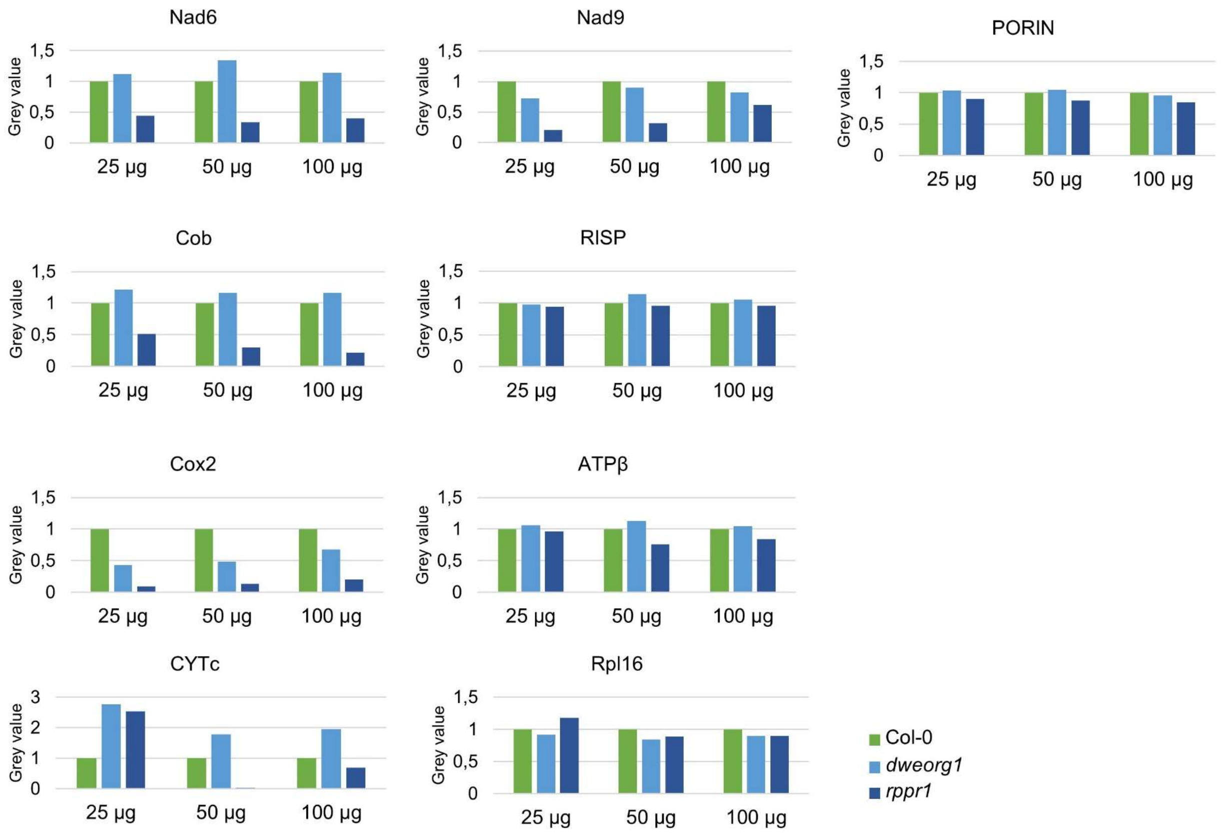

Supplementary Figure 4. Grey values of western blot. Grey values of: Nad6, Nad9, CYTc, Cob, RISP, Rpl16, Cox2, ATPβ and PORIN. Bar graphs were constructed according to the gray values of the protein bands. To determine the grey values the software ImageJ was used.

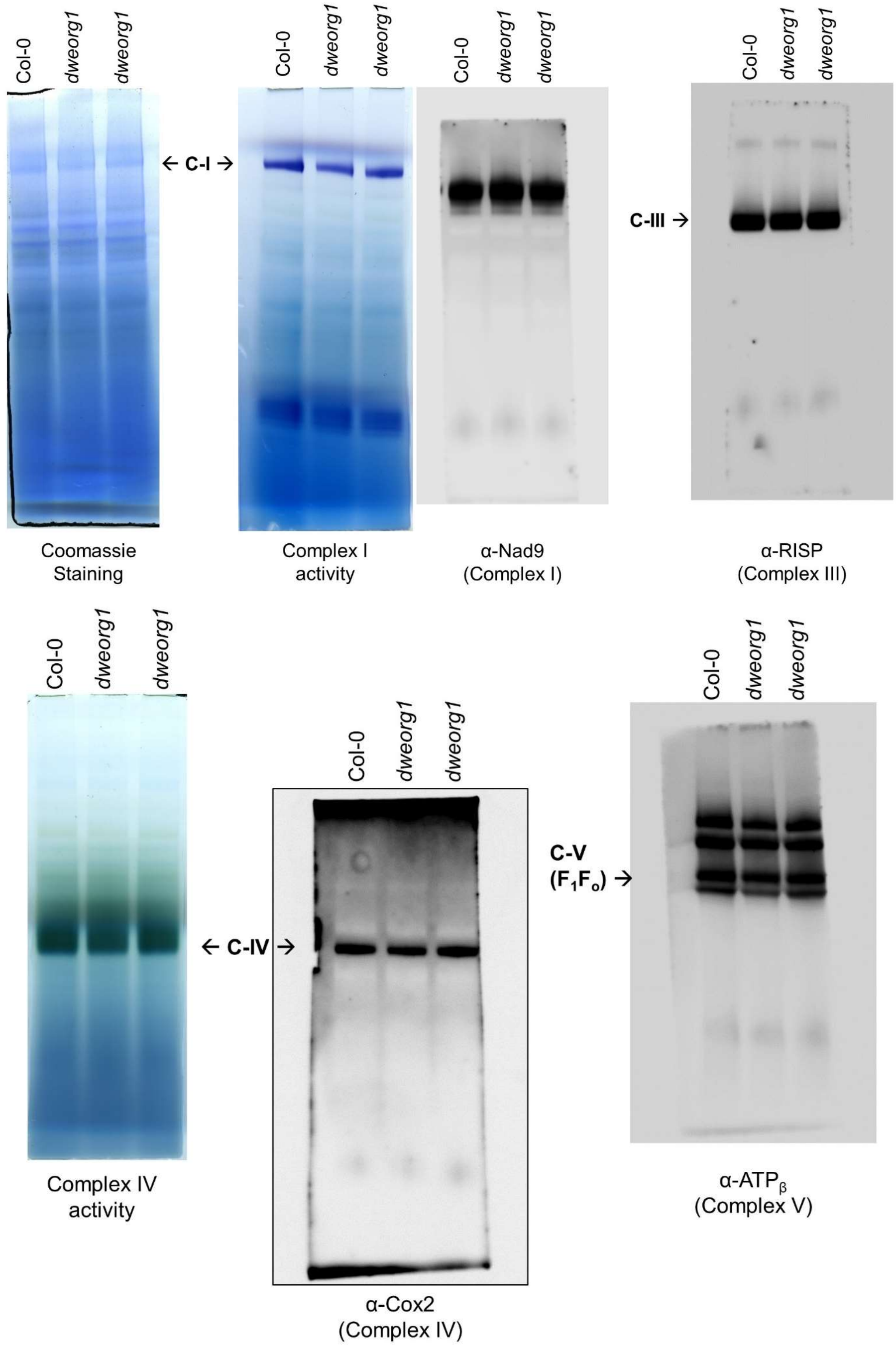

Supplementary Figure 5. Respiratory chain complexes show no obvious decrease in *dweorg1* plants. Blue native gel (BN-PAGE) analysis of mitochondrial respiratory complexes from wild type and *dweorg1* floral buds; 100 μg of crude mitochondrial extract was loaded in each lane and separated on blue native gels (4%–16% gradient gels). The NADH-dehydrogenase activity of complex I and cytochrome c oxidase activity of complex IV were stained in gel. BN-PAGE immunoblots of the respiratory complexes were performed using antibodies to the mitochondrial proteins Nad9, RISP, Cox2, and ATPβ to detect complexes I, III, IV, and V, respectively.

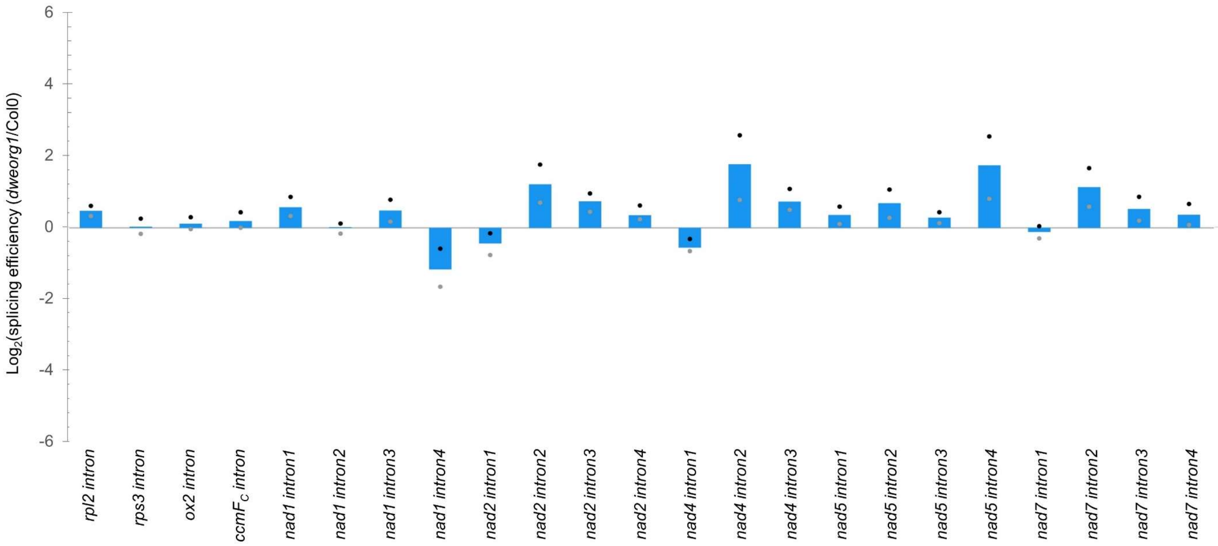

Supplementary Figure 6. The *dweorg1* plants show no remarkable defects in mitochondrial intron splicing. Quantitative RT-PCR analysis monitoring the splicing efficiencies of all mitochondrial introns in *dweorg1* relative to the wild type. Log_2 ratios of spliced to unspliced forms for each intron in mutants compared with the wild type plants are depicted. The values are means of two biological and three technical repeats with individual data points shown.

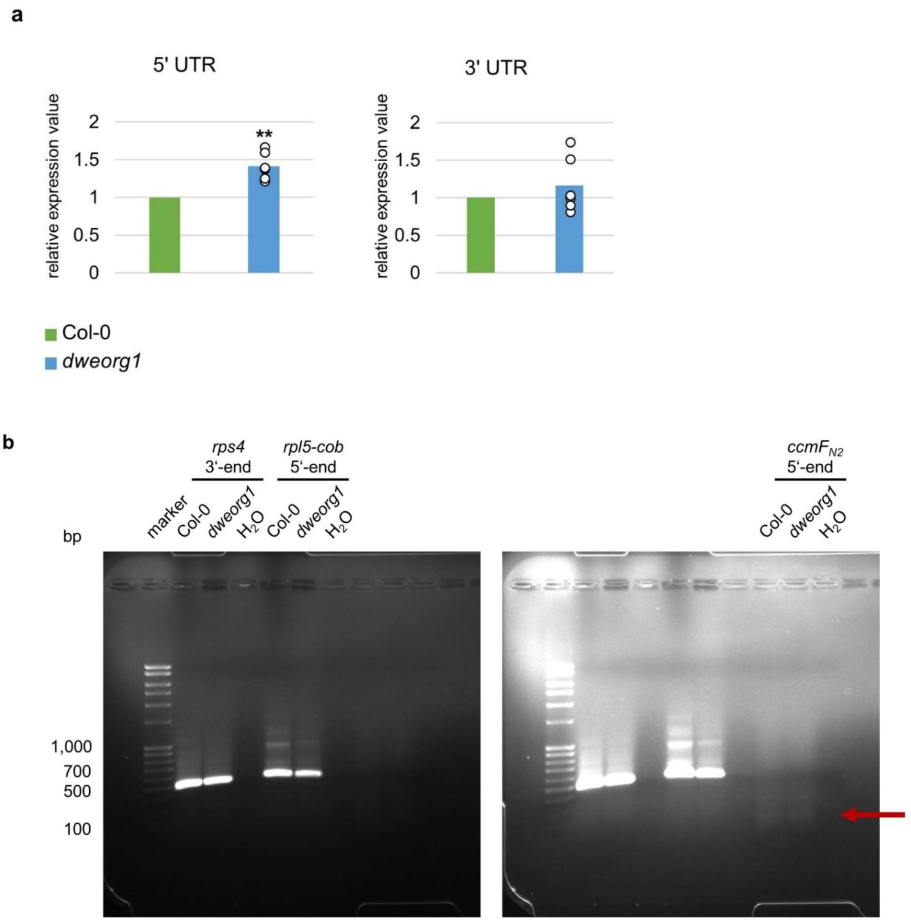

Supplementary Figure 7. Analysis of 5' and 3' termini of mitochondrial transcripts in *dweorg1* and wild type plants. (a) Level of 5' and 3' termini of *cox2* in *dweorg1* determined via quantitative RT-PCR in relation to the wild type. *ubq11* was used as an internal control. The shown values are means of three biological and two technical repeats; individual data points are shown (** $P < 0.01$, student's t-test). **(b)** Same gel with different exposure times of RT-PCR amplification of the *rpl5-cob* and *ccmF*$_{N2}$ -459 and -57 5' termini, respectively (lanes 4-9 next to the marker), and *rps4* 3' terminus (lanes 1–3 next to the marker). The red arrow indicates the faint *ccmF*$_{N2}$ PCR amplification product.

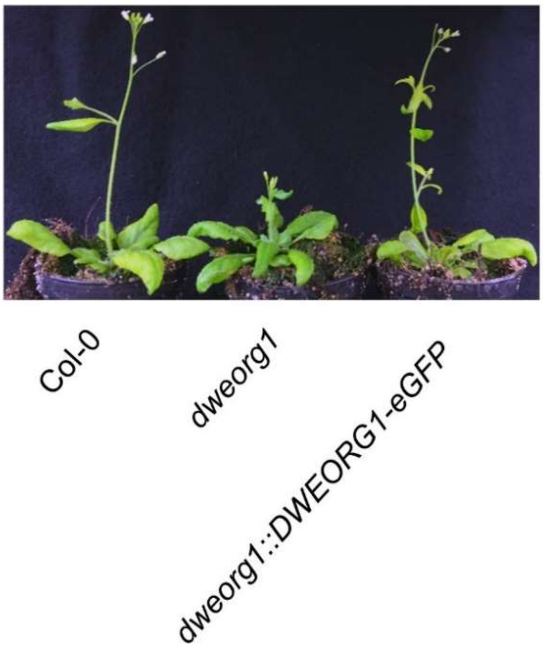

Supplementary Figure 8. Expression of a DWEORG1-eGFP fusion protein complements the *dweorg1* phenotype. Growth comparison of wild type (Col-0), *dweorg1*, and *dweorg1*-complemented with *DWEORG1-eGFP* under the control of the CaMV 35S promoter four weeks after sowing under long-day conditions (16 h light/8 h dark; 64% humidity, 22°C with a light intensity of 120–122 µmol*m^{-2}*s^{-1}).

PPR-motif		PPR-code	base
1	EVVY**R**TLLANCVLKHHVNKAEDIFNKMKELKFPT**S**	R/S	C
2	VFAC**N**QLLLLYSMHDRKKISDVLLLMERENIKP**S**	N/S	C>U>A
3	RATY**H**FLINSKGLAGDITGMEKIVETIKEEGIEL**D**	H/D	G
4	PWVC**R**SLLPLYADIGDSDNVRRLSRFVDQNP**R**	R/R	?
5	PELQ**S**ILAKYYIRAGLKERAQDLMKEIEGKGLQQ**T**	S/T	A
6	YDNC**I**SAIKAWGKLKEVEEAEAVFERLVEKYKIF**P**	I/P	?
7	MMPY**F**ALMEIYTENKMLAKGRDLVKRMGNAGIAI**G**	F/G	?
8	PSTW**H**ALVKLYIKAGEVGKAELILNRATKDNKMRP**M**	H/M	?
9	FTTY**M**AILEEYAKRGDVHNTEKVFMKMKRASYAA**Q**	M/Q	?

Supplementary Figure 9. DWEORG1 does not follow the PPR-code. Schematic illustration of DWEORG1's PPR motifs and the predicted PPR-code as described by (Yan, J. *et al.* Delineation of pentatricopeptide repeat codes for target RNA prediction. *Nucleic Acids Res.* 47, 3728–3738 (2019)).

Full-length blots/gels for cropped images

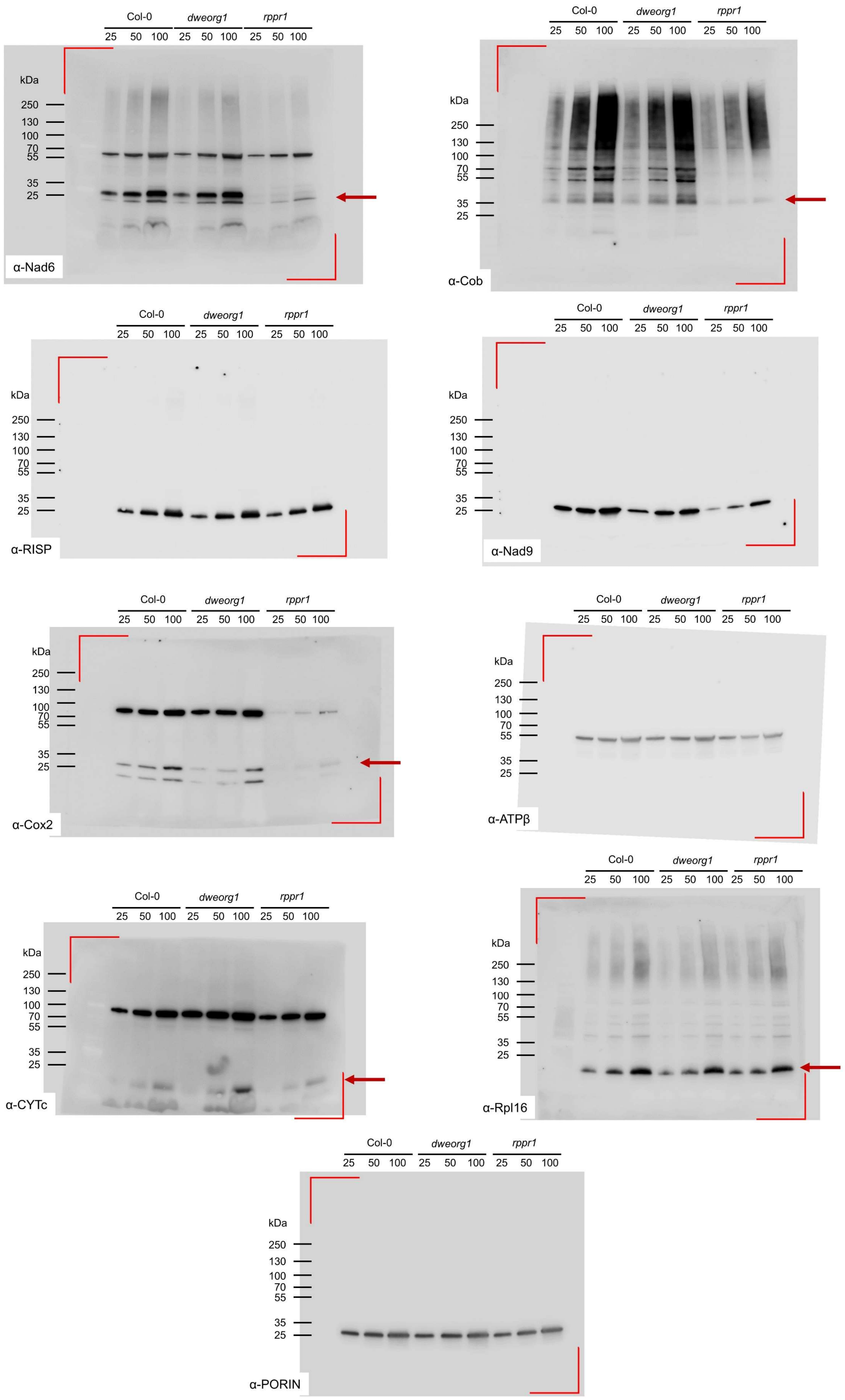

Supplementary Figure 10. Full-length blots of western blot figures shown in Figure 4a. Red lines indicate membrane edges for better visibility.

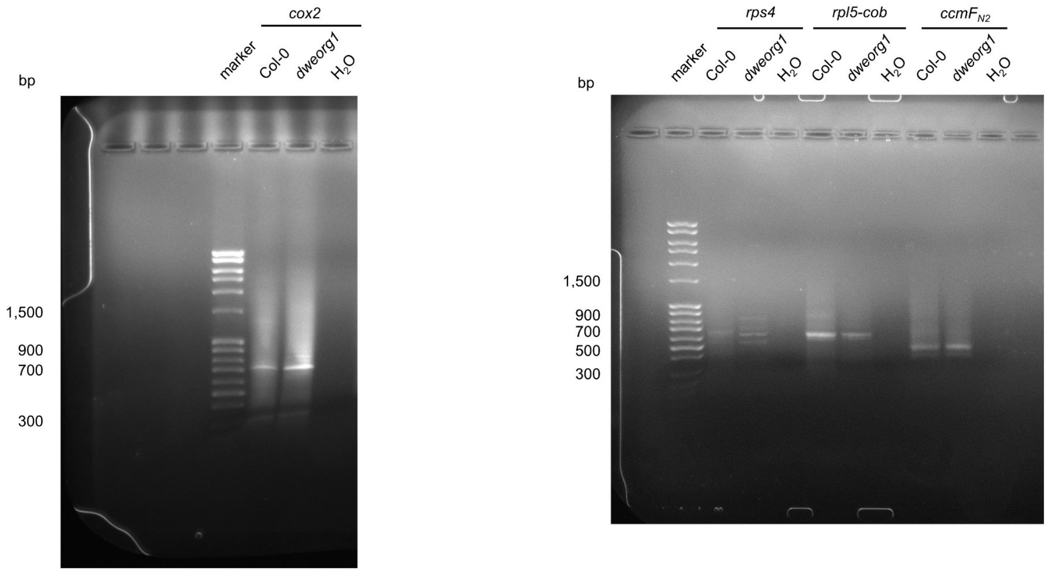

Supplementary Figure 11. Full-length gels of DNA-gels shown in Figure 4b.

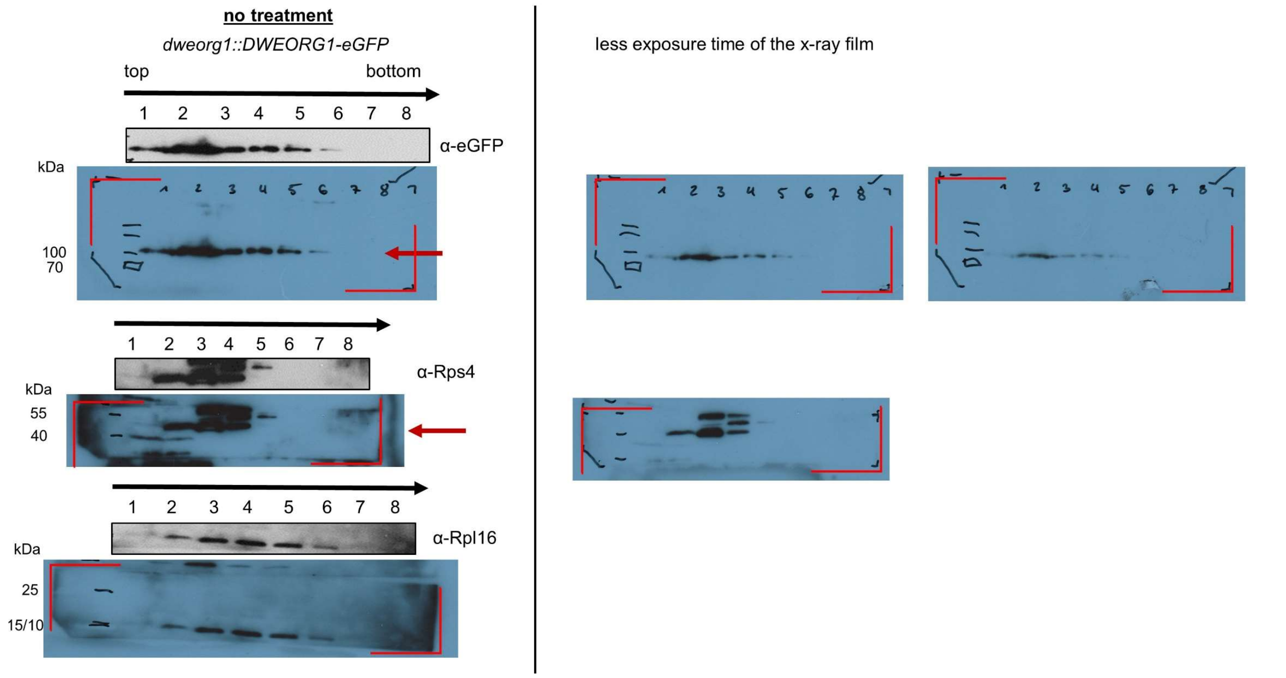

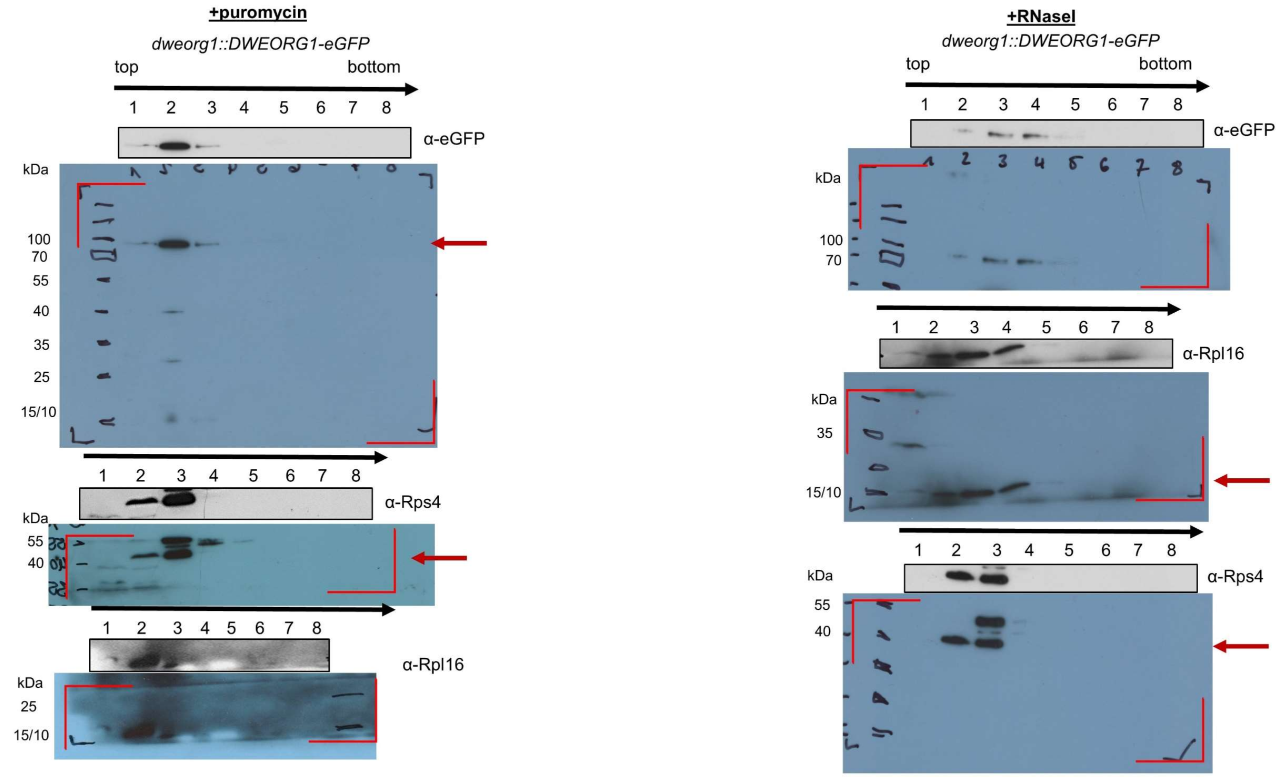

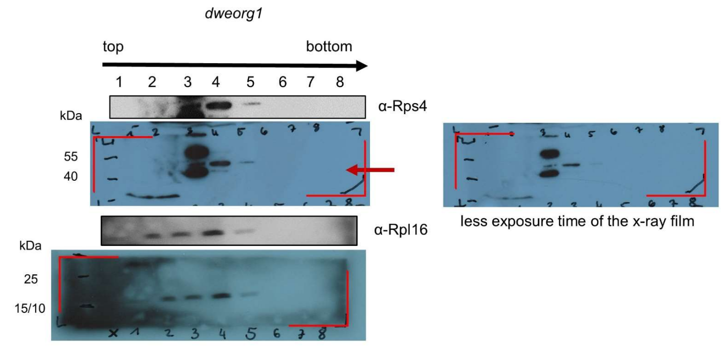

Supplementary Figure 12. Full-length blots of western blot figures shown in Figure 5a and b.

Blots were cut prior to hybridization with antibodies. Since the membrane edges are not always visible on the x-ray films red lines indicate the edges. For some membranes different exposure times are shown.

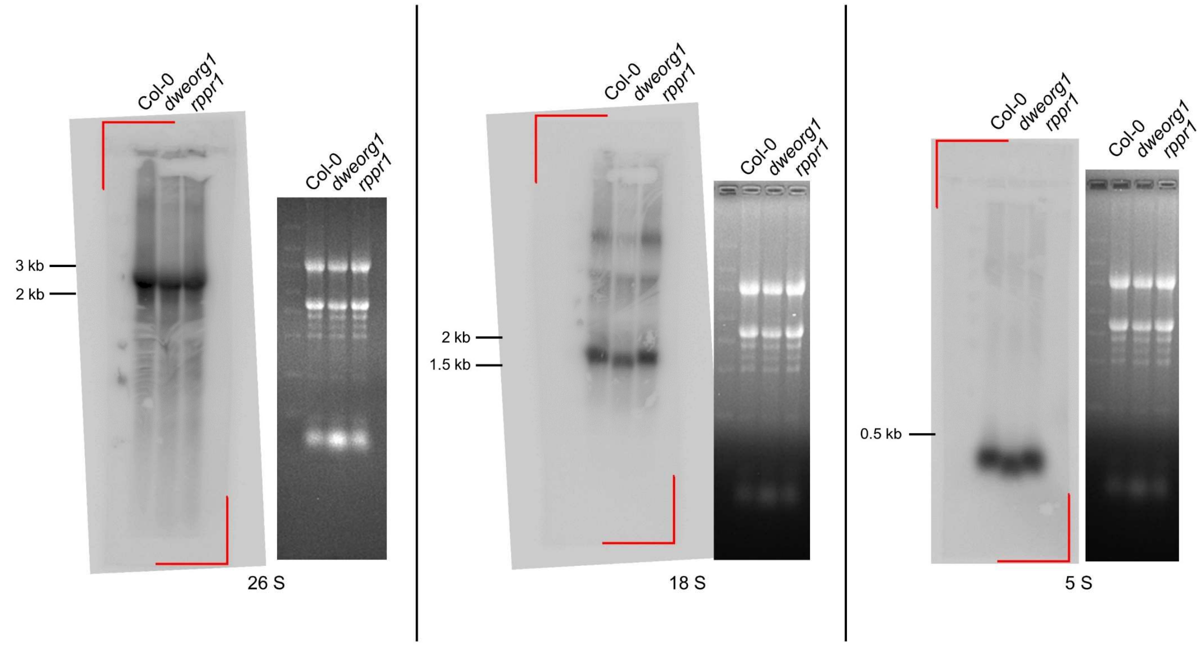

Supplementary Figure 13. Full-length blots of northern blot figures shown in Figure 6c.

Supplementary Tables

Table S1: Oligonucleotides used in this work

Description	Sequence (5'/3')	Purpose	
FK357	GATTAATTGATTGGATACCCGAGAAC	RT-PCR, unspliced *cox2* with FK550	
FK550	CTGACTATAACAGTTCCGATGAGCA	RT-PCR, unspliced *cox2* with FK357; spliced *cox2* with FK550	
FK683	ACAGCTCCAGCCGCTCACTG	RT-PCR, spliced *cox2* with FK550	
AB1088	TGGTTCACGTAGTGGGCCATCG	LB of T-DNA, screening of GK-188B06	
AB1333	ACACATTGTCTGCCTTCATCC	*DWEORG1* wild type gene, upstream of T-DNA insertion, screening of GK-188B06	
AB1334	GCAGTTTGAGTTCACAGAGGC	*DWEORG1* wild type gene, downstream of T-DNA insertion, screening of GK-188B06	
AB1453	GATAACGTGAAGGCTAAGATCC	*ubq11*, qRT-PCR, forward	
AB1454	GGAGGACCAAGTGAAGAGTAGA	*ubq11*, qRT-PCR, revers	
AB1463	TCGGCTTATCTGTCAGTCG	*atp1*_f	RT-PCR for RNA editing analysis
AB1464	GATTCCTAATGTGATGAAAGC	*atp1*_rev	
AB1465	CTTGAACTATATCATAAACTGATCT	*ccmB*_f	
AB1466	CTTGTAAACTAATCGAGACCG	*ccmB*_rev	
AB1467	TGAGAGATGTTGAAGGGG	*cob*_f	
AB1468	CTAGTAGTAAGCAATCCGCC	*cob*_rev	
AB1469	ATACCATGTTCGTATGGTGG	*cox3*_f	
AB1470	AAACCATGAAAGCCTGTTGC	*cox3*_rev	
AB1471	ACGTACATAGCTGTTCCAGC	*nad1*_f1	
AB1472	TAATTACTAGACCGACCCGC	*nad1*_rev1	
AB1473	GCCTTTCTAGGAGCATTACG	*nad1*_f2	
AB1474	TTAAGGAAGCCATTGAAAGG	*nad1*_rev2	

AB1475	GAGACAAAGAAAGATCAAGGC	*nad4*_f1
AB1476	AGCAATGATCTTCTTTAGATCG	*nad4*_rev1
AB1477	ATGTTAGAACATTTCTGTGAATG	*nad4*_f2
AB1478	CTCCTATAATAATGAACATAGGGA	*nad4*_rev2
AB1479	GAACATACAGGGAATTGGAG	*nad4*_f3
AB1480	CCAACAAGAAAAGGTATAAATATG	*nad4*_rev3
AB1483	TCATTACGAAGATGTATCACG	*rpl5*_f
AB1484	AGTCTCATCTTGTGTGTTGG	*rpl5*_rev
AB1943	CAACCCGAGATGTTAGAAGG	*atp9*_f
AB1944	TTGCTTTATGAGACTGAATGG	*atp9*_rev
AB1945	AGCGTGTCGTTCGTAATG	*ccmF*$_{N2}$_f
AB1946	ATACGTTCTACGGACCGATG	*ccmF*$_{N2}$_rev
AB1947	ACAAGAAACAACCACCAGCG	*ccmF*$_{N1}$_f1
AB1948	TCCTTTCAGGATAAAGCGGC	*ccmF*$_{N1}$_rev1
AB1949	GAAGGAACCGGACTTTTGAC	*ccmF*$_{N1}$_f2
AB1950	CGGGCTTCTATTGCTACG	*ccmF*$_{N1}$_rev2
AB1951	GAGAAGCCAAACTCGAACGG	*ccmF*$_{C}$_f1
AB1952	CGCACATAAGCCGCCGGGTCGC	*ccmF*$_{C}$_rev1
AB1953	CCACCCCAGCAGTAATGGTG	*ccmF*$_{C}$_f2
AB1954	GTGCCTTGACGCGCCGCAGCCAC	*ccmF*$_{C}$_rev2
AB1955	GGGTTGAAGTTTAGACCGC	*matR*_f1
AB1956	CTATCTCCTGATCGAGCTTG	*matR* _rev1
AB1957	GGATCGAGGTATCATTAGCC	*matR* _f2
AB1958	CTTGTTCAGGTCTTGACCG	*matR* _rev2
AB1959	CGGAATTTTCACCTATTTTTATC	*nad3*_f
AB1960	GAAGCACCCCTTTTCCATTC	*nad3*_rev
AB2024	GCCACTTCCCGTTCAGTTGCTG	*tp4*_f
AB2025	CCGCTCTTCTGAGAGCATGAAC	*atp4*_rev
AB2026	TCAAAGACCAGAAGCTACGC	*ccmC*_f
AB2027	CAAGATACGGGTTGAGAAGG	*ccmC*_rev
AB2028	GAAAGCAGAATTCGTTCGG	*nad2*_f1
AB2029	CCCACATATGAAAAGGAACTG	*nad2*_rev1
AB2030	GTTACAGCATTCCTTTCTATTGC	*nad2*_f2
AB2031	GATATGAACTGAGTGCCATTTG	*nad2*_rev2
AB2034	CATCCATAAGTAGCTTGGTCC	*nad5*_f
AB2035	CGATGACCCTCTTTAGATCG	*nad5*_rev
AB2112	GGACATCAATGGTATCGGAC	*cox2*, qRT-PCR, forward
AB2113	GAGTTTTGGCTGGTACAACC	*cox2*, qRT-PCR, revers
RS3133	TACCGAGGCTCCTCTTAACC	*act2*, qRT-PCR, forward
RS3134	AGCTTGGATGGCGACATACA	*act2*, qRT-PCR, revers
SG3176	ATACCATTGATGTCCAATAGC	cDNA synthesis for cRT analysis of *cox2*
SG3177	AATGGCTGGATCTACTACTACCTCGTCCATTG	cRT analysis, 5'/3'*cox2,* oligo1
SG3178	AATTAATCCCCCAAACCGGGGAAG	cRT analysis, 5'/3'*cox2,* oligo2
SG3233	TGGGGGATTAATTGATTGGA	qRT-PCR, *cox2*spliced with SG3234; *cox2*unspliced with SG3235
SG3234	TGATGCTGTACCTGGTCGTT	qRT-PCR, *cox2*spliced with SG3233
SG3235	AGCAGTACGAGCTGAAAGGC	qRT-PCR, *cox2*unspliced with SG3233
SG3236	GGCTATAAGTAGGCCGTTTG	*cox2* 3' end, qRT-PCR, oligo1
SG3237	CCTCGTCTTGCTTGCTTCTG	*cox2* 3' end, qRT-PCR, oligo2
SG3238	CTGCTGCTTTTAGGAGAGTG	*cox2* 5' end, qRT-PCR, oligo1
SG3239	CCCCAAACAAAGAGAGACTG	*cox2* 5' end, qRT-PCR, oligo2
SG3250	TCAGCATGCCGCGGTGAATATG	18S rRNA mitochondria, qRT-PCR, oligo1
SG3251	TGCTTCGGGCGAAACCAATTCC	18S rRNA mitochondria, qRT-PCR, oligo2
SG3252	TGCGAGGTGGGTAGTTTATC	26S rRNA mitochondria, qRT-PCR, oligo1
SG3253	GGCAGGCTTATACCATTACG	26S rRNA mitochondria, qRT-PCR, oligo2
SG3254	GCTGAAACTTAAAGGAATTGACG	18S rRNA cytosol, qRT-PCR, oligo1
SG3255	CAGTCTGTCAATCCTTACTATG	18S rRNA cytosol, qRT-PCR, oligo2
SG3256	CTGTCTACTATCCAGCGAAAC	25S rRNA cytosol, qRT-PCR, oligo1
SG3257	CCTACACCTCTCAAGTCATTTC	25S rRNA cytosol, qRT-PCR, oligo2

SG3258	CTATAACGGTCCTAAGGTAGCG	23S rRNA chloroplast, qRT-PCR, oligo1
SG3259	CTATTTCACCGAGCCTCTCTC	23S rRNA chloroplast, qRT-PCR, oligo2
SG3260	GCGTAGAGATCGGAAAGAAC	16S rRNA chloroplast, qRT-PCR, oligo1
SG3261	GGGTATCTAATCCCATTCGC	16S rRNA chloroplast, qRT-PCR, oligo2
SG3280	AATCCCCATGAAAAAGGGGC	cDNA synthesis for cRT analysis of *rps4*
SG3281	CTCTGTTCCGAACATTTCCTG	cRT analysis, 5'/3'*rps4,* oligo1
SG3282	TAGGTCATATCCCTCACGAC	cRT analysis, 5'/3'*rps4,* oligo2
SG3283	TTCGAATTGGTTCCTGGTCC	cDNA synthesis for cRT analysis of *ccmF*$_{N2}$
SG3284	TCCTGATCAGAGACCACTGTGTTCGC	cRT analysis, 5'/3'*ccmF*$_{N2}$*,* oligo1
SG3285	AGCAGGCATCGGTCCGTAGAACG	cRT analysis, 5'/3'*ccmF*$_{N2}$*,* oligo2
SG3286	CACACGATCCAGGAACTTCC	cDNA synthesis for cRT analysis of *rpl5-cob*
SG3287	TTTGAGCAACGGATCTTGACG	cRT analysis, 5'/3'*rpl5-cob,* oligo1
SG3288	ATTCTGGGACGAGTTGGAAG	cRT analysis, 5'/3'*rpl5-cob,* oligo2
SG3301	TCAGAAGAGTGCTCCCTACG	*ccmF*$_{N2}$ 5'UTR for
SG3302	ACAAAGAGGCGAAGTAGTGG	*ccmF*$_{N2}$ 5'UTR rev
SG3303	AGATCGGCGTAGTCACTCATAG	*rps4* 3'UTR for
SG3304	AGATCGTCCTTGGTCCTTTTG	*rps4* 3'UTR rev
SG3305	GAAGACGCGTGCTTTGTTTG	*rpl5* 5'UTR for
SG3306	CCATAACGTTGGCGTGATTC	*rpl5* 5'UTR rev
mitorrn18Sfull-f	ATCATAGTCAAAAGAAGAGTTTGAT	RNA gel blot hybridization of mitochondria 18S rRNA
mitorrn18Sfull-re	GGATTCAATCCAGCCACAGGTTCC	
mitorrn5Sfull-f	AAACCGGGCACTACGGTGAGACGTG	RNA gel blot hybridization of mitochondria 5S rRNA
mitorrn5Sfull-re	TCACCGGGCTTGGACCATGTCTCC	
mitorrn26Sfull-f	GTCAAGCGCACTCACTCTAACGGC	RNA gel blot hybridization of mitochondria 26S rRNA
mitorrn26Sfull-re	AATTAGGGTGCGGCAACTAAAGAGG	

Table S2: Editing status of mitochondrial transcripts analyzed by RT-PCR

Gene	Size (bp)	Predicted sites in		State of editing site in *dweorg1* plants		
		amplified region	complete gene	edited	not/not fully edited	sum
ATP1	436	4	5	4	0	4
ATP4	681	8	8	9	0	9
ATP9	272	3	4	3	0	3
CCMB	601	36	39	0	0	0
CCMC	591	25	28	0	0	0
CCMF$_{C}$	832	10	16	0	0	0
	651	6		1	0	1
CCMF$_{N1}$	480	11	22	5	0	5
	578	9		5	0	5
CCMF$_{N2}$	493	12	12	10	0	10
COB	800	6	7	6	0	6
COX2	780	12	15	12	0	12
COX3	463	7	8	6	0	6
MATR	412	4	9	1	0	1
	348	5		6	0	6
NAD1	384	5	24	4	0	4
	581	17		16	0	16
NAD2	745	17	32	12	0	12
	714	14		11	0	11
NAD3	360	8	12	7	0	7
NAD4	441	6	32	6	0	6
	475	15		14	0	14
	419	6		7	0	7
NAD5	666	16	27	17	0	17
RPL5	490	5	10	3	0	3

Table S3: Summary of SNaPshot-analysis results for the editing status of *dweorg1* mitochondrial transcripts

Shown are the edited positions in *dweorg1* transcripts that correspond to wild type (Col-0) editing positions (shown in first row of each PCR product), editing sites are identified by the number of nucleotides from the ATG. Primers of staggered lengths of 3–5 nucleotides, specific to each editing position were used (shown in second row of each transcript). Grey positions were not captured or could not be analyzed. Red positions were not captured in the SNaPshot-anaylsis but were confirmed by sequencing of PCR-products.

PCR product no.	Pool no.	PCR product name	Editing position/Primer											
1	1	atp1a	1110	1178	1292									
1	1	atp1a	26R	41F	57F									
2	5	atp1b	1415	1484										
2	5	atp1b	50R	41F										
3	1	atp4a	89	138	215	248								
3	1	atp4a	41R	34R	30F	34F								
4	7	atp4b	250	251	395	416								
4	7	atp4b	41F	38R	56F	44R								
5	11	atp9a	53	83										
5	11	atp9a	39F	42R										
6	2	atp9b	167	224										
6	2	atp9b	53R	56F										
7	3	ccmF$_{N2}$a	65	176	208		93							
7	3	ccmF$_{N2}$a	26F	30F	26R									
8	2	ccmF$_{N2}$b2	320	344										
8	2	ccmF$_{N2}$b2	53F	50R										
8	6	ccmF$_{N2}$b1	226	259	277	356	391							
8	6	ccmF$_{N2}$b1	53F	38F	26R	34F	30R							
9	1	ccmBa2	137	159	164	193								
9	1	ccmBa2	44F	47F	48R	60R								
9	4	ccmBa3	154	179	181	286	71	28						
9	4	ccmBa3	56F	26F	34R	26R	38R	59R						
9	19	ccmBa1	16			80	128	148	149	172	194			
9	19	ccmBa1	47F			41R	41F	44F	44R	47R	50R			
10	2	ccmBb2	367	406	475	485		569		476	494			
10	2	ccmBb2	47F	41R	50F	44R		47R						
10	15	ccmBb3	554	566	596									
10	15	ccmBb3	38F	44R	50R									
10	18	ccmBb1	304	338	379	380	424	428	467	512	551	576		
10	18	ccmBb1	26F	30F	34F	26R	38F	30R	41F	44F	47F	41R		
11	3	ccmCa	103	133	184	262	331	395		179				
11	3	ccmCa	47R	50R	53F	56F	53R	50F						
12	2	ccmCb1	400	421	436	463	497	521	548	568	624	656		
12	2	ccmCb1	26F	30F	26R	30R	34R	34F	38F	38R	41F	44F		
12	4	ccmCb2	446	458	575	614	618	673						
12	4	ccmCb2	47F	41R	34F	53F	44R	47R						
12	5	ccmCb3		467	608	619								

12	5	ccmCb3		44R	38F	56R								
13	3	ccmF$_{N1}$a1	44	104	157	262								
13	3	ccmF$_{N1}$a1	30R	34R	38R	38F								
13	19	ccmF$_{N1}$a2	143	269										
13	19	ccmF$_{N1}$a2	59R	53F										
14	6	ccmF$_{N1}$b	289	378										
14	6	ccmF$_{N1}$b	57R	60R										
15	7	ccmF$_{N1}$c	709	710	779									
15	7	ccmF$_{N1}$c	26F	26R	30F									
16	6	ccmF$_{N1}$d	791	806	955									
16	6	ccmF$_{N1}$d	56F	51R	26F									
17	3	ccmF$_{C}$a	50	103	122	146	155		123	175	333			
17	3	ccmF$_{C}$a	41R	41F	44F	47F	44R							
18	4	ccmF$_{C}$b	160	334	406	415								
18	4	ccmF$_{C}$b	30F	38F	41F	30R								
19	11	ccmF$_{C}$c	1172	1215										
19	11	ccmF$_{C}$c	56F	59F										
20	4	ccmF$_{C}$d	1246	1280	1327									
20	4	ccmF$_{C}$d	53R	50R	56R									
21	5	cox2a2	24	71	138									
21	5	cox2a2	34F	44F	47R									
21	6	cox2a1	24	27										
21	6	cox2a1	30F	34R										
22	2	cox2b	278	379		253								
22	2	cox2b	56R	59R										
23	8	cox2c	476	557	581									
23	8	cox2c	30F	50F	30R									
24	17	cox2d	698	721	742									
24	17	cox2d	41F	44F	54R									
25	5	cox3a	245	257	314	413	422							
25	5	cox3a	47F	34R	30R	50F	26R							
26	19	cox3b	112	311										
26	19	cox3b	56R	53R										
27	9	cyta	286	325	118									
27	9	cyta	26F	30R	47F									
28	7	cytb	568	853										
28	7	cytb	34R	47F										
29	16	cytc	908	982	1084									
29	16	cytc	44F	50F	53R									
30	13	matRa	374	461										
30	13	matRa	38F	26F										
31	10	matRb	1730	1731	1751									
31	10	matRb	50F	56R	47R									
32	12	matRc	1771	1807	1895									
32	12	matRc	44R	41R	50R									
33	5	nad1a	167	265	307	308								
33	5	nad1a	26F	38R	30F	41R								

34	18	nad1b1	492	500	536	571								
34	18	nad1b1	50F	34R	53R	59R								
34	19	nad1b2	490	493										
34	19	nad1b2	26F	38R										
35	10	nad1c1	580	635	674	725	743	937						
35	10	nad1c1	30F	26R	30R	26F	34R	44F						
35	11	nad1c2	755	823	898	928								
35	11	nad1c2	34F	41F	44F	38R								
36	1	nad2a	59	89	90	344	389	394						
36	1	nad2a	26F	51F	51R	54F	38F	57R						
37	7	nad2b	400	427	461	530	558	695						
37	7	nad2b	30R	41R	47R	50F	44F	53F						
38	6	nad2c1	821	842	953	961	1091	1160	1279	1280	1309			
38	6	nad2c1	41F	48R	44F	38R	47F	54R	50F	45R	41R			
38	10	nad2c2	991	995										
38	10	nad2c2	34F	50R										
39	3	nad2d	1433	1436	1490									
39	3	nad2d	34F	56R	59R									
40	16	nad3a1	8	26		149	211	212	250					
40	16	nad3a1	34F	38R		30F	56F	53R	47R					
40	19	nad3a2	64	83										
40	19	nad3a2	50F	26R										
41	10	nad3b	254	347	352									
44	10	nad3b	38F	1033	53R									
42	1	nad4a2	124	164	197		608	659	766	767	784	836	896	
42	1	nad4a2	38R	44R	54R									
42	9	nad4a1	29	74	84	107	158	166	317	362	376	403		
42	9	nad4a1	26R	30F	34R	47R	34F	41R	53F	56F	56R	59R		
43	12	nad4b	436	449		437								
43	12	nad4b	38F	56R										
44	8	nad4c	1033	1101	1129	1172	1355	1373	977	1148				
44	8	nad4c	38F	41F	47R	44F	47F	41R	34F	53F				
45	8	nad4La2	55	95	110									
45	8	nad4La2	26R	59F	38R									
45	19	nad4La1	41	86	100	131	158							
45	19	nad4La1	34F	30F	30R	38F	34R							
46	9	nad4LB	188	197										
46	9	nad4LB	50F	50R										
47	9	nad5a	155	272	358	374	398		242					
47	9	nad5a	38F	41F	44F	44R	38R							
48	11	nad5b1	494	548	553		608	609	676	725	764	835		
48	11	nad5b1	30R	53F	47R		47F	34R	50R	30F	53R	56R		
48	12	nad5b2		629	713	598								
48	12	nad5b2		47R	36R	59R								
49	18	nad5c	863	875	1275									
49	18	nad5c	53F	44R	38R									
50	4	nad5d2	1580	1895										

50	4	nad5d2	44F	50F										
50	10	nad5d1	1550		1610		1916	1918	1958					
50	10	nad5d1	47F		44R		53F	41R	38R					
51	12	nad6a1	26	53	88	89	161	169						
51	12	nad6a1	41F	44F	26F	32R	53F	53R						
51	13	nad6a2	95	103										
51	13	nad6a2	30F	26R										
52	14	nad6b	191	446	463									
52	14	nad6b	47R	26F	59R									
53	12	nad7a1	24	77	137	200	213	251						
53	12	nad7a1	47F	31F	35F	50F	28R	24R						
53	13	nad7a2	209	244	316	335								
53	13	nad7a2	50R	41R	44F	44R								
54	14	nad7b	344	578										
54	14	nad7b	53F	56F										
55	13	nad7c1	739	769	795	926	963	1057	1103	1124	1137			
55	13	nad7c1	29R	32R	53R	41F	36R	47F	50F	53F	56R			
55	15	nad7c2		575	679									
55	15	nad7c2		44F	50F									
56	14	nad9a	92	167	190	298	328							
56	14	nad9a	47F	50F	34R	38R	41R							
57	18	nad9b	398	439										
57	18	nad9b	47R	50R										
58	14	orfxa1	59	97	144	145	173	361	364	406	407		387	586
58	14	orfxa1	56R	34F	38F	26R	30R	44F	44R	30F	50R			
58	16	orfxa2	161	164	377	379								
58	16	orfxa2	41F	34R	47F	41R								
59	15	orfxb1	409	412	440	505		538	581	587	643	649	693	
59	15	orfxb1	26F	34R	38R	41F		26R	34F	30R	30F	41R	47R	
59	17	orfxb2	474	530	665	666	700	746						
59	17	orfxb2	47F	60F	50F	47R	60R	57R						
60	9	rpl5a	58	59										
60	9	rpl5a	59F	53R										
60	16	rpl5a	35	47	64	92								
60	16	rpl5a	38F	26R	30R	50R								
61	17	rpl5b	169	317	329	512								
61	17	rpl5b	51R	38F	30R	44R								
62	7	rpl16a	30	34	61	209								
62	7	rpl16a	34F	53R	56R	38F								
63	18	rpl16b	440	506	512									
63	18	rpl16b	56F	59F	56R									
64	8	rps3a	64	603										
64	8	rps3a	34R	26F										
65	11	rps3b	1470	1534	1598									
65	11	rps3b	26R	50F	26F									
66	14	rps3c	1344	1352										
66	14	rps3c	41F	53R										

67	17	rps4a	77	88	175	226	235	299	308					
67	17	rps4a	26F	41R	57F	30F	34R	34F	38R					
68	16	rps4b	332	377	524									
68	16	rps4b	56F	26F	44R									
69	17	rps4c	956	967										
69	17	rps4c	53F	26R										
70	13	rps4d1	992	1042	1043									
70	13	rps4d1	56F	34F	47R									
70	15	rps4d2	1052	1057										
70	15	rps4d2	53F	53R										
71	8	rps12a	84	104	146	196	221	269						
71	8	rps12a	53R	44R	50R	56F	56R	59R						
72	15	rps12b	284	285										
72	15	rps12b	47F	56R										
73	1	atp6	475											
73	1	atp6	30R											
74	5	orf114	309											
74	5	orf114	56F											
75	15	orf240	199											
75	15	orf240	56F											
76	20	ndhBa	149	467	585	746								
76	20	ndhBa	26F	26R	30F	30R								
77	20	ndhBb	830	836	872	1255	1481							
77	20	ndhBb	34R	38R	41R	44R	34F							
78	20	ndhDa	2	383										
78	20	ndhDa	47R	38F										
79	20	ndhDb	674	878	887									
79	20	ndhDb	41F	44F	50R									
80	20	psbEF	1B	1F										
80	20	psbEF	53R	47F										
81	20	atpF	92											
81	20	atpF	50F											
82	4	accD	749											
82	4	accD	59F											
83	11	clpP	559											
83	11	clpP	59R											
84	3	matK	706											
84	3	matK	59F											
85	6	ndhF	290											
85	6	ndhF	60F											
86	16	ndhG	50											
86	16	ndhG	59F											
87	7	petL	5											
87	7	petL	50R											
88	2	rpoBa	338	551										
88	2	rpoBa	59F	47R										
89	10	rpoBb	2432											

89	10	rpoBb	56F											
90	12	rps14	149											
90	12	rps14	59F											
